# The Role of TMEM16A/ERK/NK-1 Signaling in Dorsal Root Ganglia Neurons in the Development of Neuropathic Pain Induced by Spared Nerve Injury (SNI)

**DOI:** 10.1007/s12035-021-02520-9

**Published:** 2021-08-18

**Authors:** Qinyi Chen, Liangjingyuan Kong, Zhenzhen Xu, Nan Cao, Xuechun Tang, Ruijuan Gao, Jingrong Zhang, Shiyu Deng, Chaoyang Tan, Meng Zhang, Yang Wang, Liang Zhang, Ketao Ma, Li Li, Junqiang Si

**Affiliations:** 1 Department of Physiology, The Key Laboratory of Xinjiang Endemic and Ethnic Diseases, Shihezi University Medical College, Shihezi, China; 2 Department of Anesthesiology, Xiangyang Central Hospital, Affiliated Hospital of Hubei University of Arts and Science, Xiangyang, China; 3 NHC Key Laboratory of Prevention and Treatment of Central Asia High Incidence Diseases, First Affiliated Hospital, Shihezi University School of Medicine, Shihezi, China; 4 Department of Anesthesiology, Union Hospital, Tongji Medical College, Huazhong University of Science and Technology, Wuhan, China; 5 Department of Security, Karamay Army Division, Xinjiang Uygur Autonomous Region, Chinese People’s Liberation Army, Karamay, China; 6 Department of Anesthesiology, Sichuan Academy of Medical Sciences, Sichuan Provincial People’s Hospital, Chengdu, China; 7 Department of Physiology, Medical College of Jiaxing University, Jiaxing, China; 8 Department of Physiology, School of Basic Medicine and Tongji Medical College, Huazhong University of Science and Technology, Wuhan, China

**Keywords:** Dorsal root ganglion, Neuropathic pain, TMEM16A, MEK/ERK signaling pathway, NK-1

## Abstract

Increasing evidence suggests that transmembrane protein 16A (TMEM16A) in nociceptive neurons is an important molecular component contributing to peripheral pain transduction. The present study aimed to evaluate the role and mechanism of TMEM16A in chronic nociceptive responses elicited by spared nerve injury (SNI). In this study, SNI was used to induce neuropathic pain. Drugs were administered intrathecally. The expression and cellular localization of TMEM16A, the ERK pathway, and NK-1 in the dorsal root ganglion (DRG) were detected by western blot and immunofluorescence. Behavioral tests were used to evaluate the role of TMEM16A and p-ERK in SNI-induced persistent pain and hypersensitivity. The role of TMEM16A in the hyperexcitability of primary nociceptor neurons was assessed by electrophysiological recording. The results show that TMEM16A, p-ERK, and NK-1 are predominantly expressed in small neurons associated with nociceptive sensation. TMEM16A is colocalized with p-ERK/NK-1 in DRG. TMEM16A, the MEK/ERK pathway, and NK-1 are activated in DRG after SNI. ERK inhibitor or TMEM16A antagonist prevents SNI-induced allodynia. ERK and NK-1 are downstream of TMEM16A activation. Electrophysiological recording showed that CaCC current increases and intrathecal application of T16Ainh-A01, a selective TMEM16A inhibitor, reverses the hyperexcitability of DRG neurons harvested from rats after SNI. We conclude that TMEM16A activation in DRG leads to a positive interaction of the ERK pathway with activation of NK-1 production and is involved in the development of neuropathic pain after SNI. Also, the blockade of TMEM16A or inhibition of the downstream ERK pathway or NK-1 upregulation may prevent the development of neuropathic pain.

**Supplementary Information:**

The online version contains supplementary material available at 10.1007/s12035-021-02520-9.

## Introduction

Neuropathic pain is a clinically common chronic refractory pain syndrome that affects approximately 7–10% of the global population [1, 2]. The pathogenesis of neuropathic pain is complex and lacks a clear molecular mechanism, which is a major challenge in clinical treatment.

The transmission and processing of the pain signals depend on the activity of ion channels in pain-related afferent nerve fibers. Recent studies have shown that chloride ion concentrations are increased in dorsal root ganglion neurons following nerve injury [3]. This increase enhances the excitability of dorsal root ganglion neurons. Activation of chloride channels in the sensory neurons leads to chloride outflow and depolarization due to higher chloride concentration [4, 5].

Increasing evidence suggests that chloride channels may be involved in the generation and development of neuropathic pain [6, 7]. Calcium-activated chloride channels (CaCCs) are Ca^2+^ concentration-dependent anion channels and Ca^2+^ is used as a second messenger. In 2008, TMEM16A was identified as the main functional molecule in CaCCs [8–10]. Studies have shown that the levels of CaCCs are increased after nerve injury [11]. CaCCs, especially TMEM16A, can increase the excitability of DRG neurons under inflammatory conditions and exacerbate formalin-mediated inflammatory pain [12, 13]. Recently, our studies have shown that TMEM16A plays a key role in persistent chronic constriction injury-induced hyperalgesia [14]. TMEM16A is activated by an increase in the intracellular calcium concentrations, leading to chloride efflux [15, 16]. Therefore, TMEM16A may promote the depolarization of nociceptive neurons and is a critical factor in generation of the action potentials.

ERK1/2 is one of the classic mitogen-activated protein kinase (MAPK) signaling pathways that can be activated by Ca2 + , protein kinase C (PKC), and growth factors to regulate cell activity. The activated form of ERK1/2, p-ERK1/2, stimulates the production and release of inflammatory factors and amplifies pain signals, which can also contribute to the development of neuropathic pain [17–19]. Inhibition of ERK1/2 kinase activation can reduce the symptoms of neuropathic pain, increase the effectiveness of opioids, reduce nociceptive feelings and increase the expression of antinociceptive factors. ERK1/2 regulates the expression of neurokinin-1 receptors (NK-1) [20]. NK-1 is a receptor for substance P, which is closely related to pain. Although the role of ERK activation in inflammatory and neuropathic pain has been well documented, it is unclear whether ERK activation is involved in TMEM16A-induced neuropathic pain.

Recently, the overexpression of TMEM16A in breast cancer and squamous cell carcinoma cells was reported to induce ERK1/2 phosphorylation, and knockdown of TMEM16A or use of inhibitors to reduce CaCC channel activity can reduce ERK1/2 activation [21]. After TMEM16A knockdown, the phosphorylation levels of MEK and ERK1/2 were significantly decreased, indicating that TMEM16A regulates cardiac fibrosis through the MAPK signaling pathway [22]. This result suggests that the MEK/ERK pathway plays a role in TMEM16A-mediated tumor proliferation, migration, and myocardial infarction [23, 24].

In the present study, we investigated the respective roles of TMEM16A and ERK/NK-1 signaling on DRG in neuropathic pain and the ways in which they participate in pain. These studies provide, to the best of our knowledge, the first mechanistic description of the role of TMEM16A in neuropathic pain and suggest that this protein may play an important role in facilitating the generation and development of neuropathic pain.

## Materials and Methods

### Establishment of SNI Models

Adult male Sprague–Dawley rats (180–200 g) were used according to the guidelines approved by the Animal Ethics Committee at the First Affiliated Hospital of Shihezi University School of Medicine, China (approval no. A2017-170–02). The spared nerve injury (SNI) model of neuropathic pain was established in rats according to the description published by Decosterd and Woolf [25]. Under anesthesia with 1% sodium pentobarbital (50 mg/kg i.p.), the skin on the dorsal part of the left thigh was incised, and the sciatic nerve and its three branches behind the femur were exposed through the biceps femoris muscle. The tibial nerve and common peroneal nerve were ligated with 5–0 silk, and a 2–4 mm region of the nerve was removed at the distal end of the ligation. Care was taken not to damage the sural nerve during the operation. The wound was closed by suturing layer by layer. Sham rats were subjected only to the exposure of the nerves without nerve ligation and transection. Rats with limb paralysis after the surgery were excluded.

### Intrathecal Catheter Implantation

Intrathecal catheter implantation was performed according to the previously reported method [26]. Rats were anesthetized with intraperitoneal injection of 1% pentobarbital sodium (50 mg/kg), and a 2.0 cm longitudinal incision was made above the L5-6 vertebrae; the fascia and muscle were separated, and a polyethylene catheter (PE-10) was pushed through the intervertebral space until clear cerebrospinal fluid flow was observed and then gently moved 2 cm upwards. Intrathecal implantation was verified by the lidocaine test.

### Experiment Protocol

To investigate the mechanism of action of TMEM16A in neuropathic pain in SNI rats, SD rats were randomly divided into four groups: the sham group, SNI group, SNI + 5%DMSO group, and SNI + T16Ainh-A01 (T16A) or U0126 group. T16Ainh-A01 is a specific inhibitor of TMEM16A, and U0126 is a specific inhibitor of MEK. T16Ainh-A01 and U0126 were delivered through an intrathecal catheter on day 14 after SNI operation. Behavioral tests were performed every 1 h within 8 h after administration. Since SNI increased the expression of TMEM16A and the MEK/ERK signaling pathway, we detected the changes in the expression levels of TMEM16A, p-MEK, p-ERK1/2, and NK-1 in the presence and in the absence of TMEM16A or U0126 inhibitors after intrathecal administration of T16Ainh-A01 (10 μg) or U0126 (10 μg) every 6 h five times starting from day 12 after the surgery. DRG samples were obtained on day 14 after nerve injury.

To investigate whether the antiallodynic effect of TMEM16A inhibitor is mediated by a reduction in peripheral nerve injury-induced hyperexcitability, the CaCC current and action potential were recorded in L4-L6 DRGs of sham and SNI rats in the presence and in the absence of TMEM16A inhibitor T16Ainh-A01 (20 μM).

On the other hand, in order to further confirm the relationships of TMEM16A, the MEK/ERK signaling pathway, and NK-1, SNI rats were randomly divided into 3 groups: the sham group, sham + 5%DMSO (DMSO) group, and sham + E-act group. E-act is a specific agonist of TMEM16A. E-act (10 μg) was delivered through an intrathecal catheter on day 14 after the operation, and then repeated the above experiment.

### Behavioral Tests

Pain behavioral tests were performed 1 day prior to the surgery (baseline conditioning), 1, 2, and 3 weeks after the surgery, and 5 min before the administration and every hour within 8 h after the administration. Rats were acclimatized to the environment for 30 min before testing. On any given day, the order of tests was randomized, and successive tests were interrupted by a 5–10 min rest interval.

Thermal sensitivity was assessed by measuring the thermal withdrawal latency (TWL) of the hind paw in response to radiant heat. The rats were placed in a transparent plexiglass box, and a radiant heat source was focused on the plantar surface of the left hind paw. A digital timer automatically recorded the duration between stimulation initiation and the tested paw lift. Cold allodynia was determined by cold stimulation of acetone-induced paw withdrawal duration in rats. Absolute acetone (100 μl) was used to place the plantar surface of the left hind paw, and the duration of paw withdrawal after acetone application was recorded. As a control, 100 μl of water at room temperature was used before and after the acetone test. The withdrawal response was not induced by water. Mechanical allodynia was evaluated using a dynamic plantar esthesiometer (Ugo Basile, Stoelting, IL, USA), an automated version of the von Frey hair. Rats were placed in plexiglass boxes on an elevated metal grid. Then, a straight metal filament (0.5 mm in diameter) was focused on the plantar surface of the left hind paw, and a mechanical stimulus was applied by an automated testing device. The force-induced withdrawal response was automatically recorded when the rat withdrew its hind paw.

### Western Blotting

According to a previously reported protocol [27], L4-L6 DRGs were removed after the rats were euthanized. DRGs were dissected and homogenized in RIPA lysis solution (Millipore, Billerica, MA, USA, # 20–188) containing 1:100 protease inhibitor cocktail (Rockford, IL, USA, cat# 78,410) and 1:100 phosphatase inhibitor cocktail (Pierce, Thermo Fisher Scientific Inc., and cat# 78,420), and the homogenate was centrifuged at 14,000 × *g* for 15 min. The supernatant was aspirated. Protein concentration was determined using a bicinchoninic acid (BCA) kit. The protein sample was separated by 10% SDS-PAGE and transferred onto a PVDF membrane (Millipore, Billerica, MA, USA). After incubation with blocking buffer for 2 h at room temperature, the membrane was incubated with primary antibodies against TMEM16A (1:1,000, ab53212; Abcam, Cambridge, MA, USA), p-MEK (1:1,000, Cell Signaling Technology, USA), MEK (1:1,000, Cell Signaling Technology, USA), p-ERK1/2 (1:1,000, Cell Signaling Technology, USA), ERK1/2 (1:1,000, Cell Signaling Technology, USA), NK-1 (1:500, Santa Cruz Biotechnology, Inc. Dallas, TX, USA), or beta-actin (1:1,000; Santa Cruz Biotechnology, Santa Cruz, CA, USA) overnight at 4 °C. After washing of the primary antibody with TBST, the membrane was incubated in the presence of secondary horseradish peroxidase-conjugated goat anti-mouse or goat anti-rabbit IgG antibodies (1:10,000 or 1:20,000, respectively, Santa Cruz Biotechnology, Santa Cruz, CA, USA) for 2 h at room temperature; then, the membrane was washed with TBST. The membrane was developed by enhanced chemiluminescence (GE Healthcare, Chicago, IL, USA). Band intensities were quantified by Image-Pro Plus 6.0 software (Media Cybernetics, Rockville, MD, USA) and normalized against a loading control (beta-actin).

### Immunohistochemistry

The rats were deeply anesthetized by intraperitoneal injection of 1% sodium pentobarbital. Aortic perfusion was performed with 0.9% saline and 4% paraformaldehyde, and left L4-L6 DRG was quickly removed. Extracted DRGs were placed in 4% paraformaldehyde at 4 °C overnight and dehydrated in 30% sucrose solution for 4 h. Dehydrated DRGs were placed in OCT embedding agent and sectioned at 5 μm thickness. Frozen sections of DRGs were incubated for 2 h in PBS with 0.3% Triton X-100 (Fluka, Spain) and 10% bovine serum albumin (Biological Industries, Israel) and overnight at 4 °C with primary antibodies. The following primary antibodies were used: rabbit anti-TMEM16A (1:100; Abcam ab25117, Cambridge, MA), rabbit anti-p-ERK1/2 (1:100; Santa Cruz, Dallas, TX), and rabbit anti-NK-1 (1:100, Santa Cruz). The following cell-specific markers were used: mouse anti-neurofilament-200 (NF-200, a marker for myelinated A-fibers, 1:100, Chemicon, Billerica, MA), FITC-conjugated isolectin B4 (IB4, a marker for unmyelinated nonpeptidergic C-fibers, 20 μg/mL, Sigma, St. Louis, MO), and mouse anti-CGRP (a marker for peptidergic C-fibers, 1:200; Abcam, USA). The sections were rinsed with PBS, and fluorescein isothiocyanate-labelled goat anti-mouse or goat anti-rabbit secondary antibodies were incubated for 2 h at room temperature. For single immunofluorescence staining, the sections were treated with PI (1:1,000) for 2 min and washed with PBS. All sections were finally sealed with 75% glycerol and imaged using a confocal laser scanning microscope (LSM710; Carl Zeiss AG, Oberkochen, Germany). DRG cells were divided into two types according to the diameter of DRG neurons: large neurons over 40 μm and medium-sized/small neurons less than 40 μm. The selected neurons had the following characteristics: the nucleus was in the center, and the cell membrane was intact. The fluorescence intensity of the DRG neurons was evaluated by Image-Pro Plus 6.0 software.

### Intact DRG Preparation and Electrophysiological Recording

According to a previously described method [6], the entire DRG was placed in artificial extracellular fluid maintained at 4 °C and supplemented with (in mM): 137 NaCl, 5.9 KCl, 2.2 CaCl_2_, 1.2 MgCl_2_, 10 HEPES, and 4 D-glucose. Using a glass dropper, DRG was repeatedly bathed for 2–4 min at 37 °C in a digestive solution containing 0.24 mg/mL type III trypsin (Sigma) and 0.6 mg/mL type A collagenase (Sigma). After digestion, the cell suspension was placed into a cell culture dish, and the cells were allowed to adhere to the surface. After the cells completely adhered, oxygen-saturated extracellular fluid was added. For electrophysiological recording, a 100 × magnification microscope (Nikon Eclipse Ti, Tokyo, Japan) was used to select DRG cells with a smooth membrane surface and good translucency. Pipettes (5–10 MΩ) and a MultiClamp 700B amplifier (Axon Instruments, America) were used to record the whole-cell ClCa current at 23–25 °C.

In the voltage-clamp mode, the voltage was gradually adjusted to the test voltage (− 100 to + 100 mV) in 20 mV increments for 1000 ms. At this point, the extracellular solution contained (in mM) 137 NaCl, 5.9 KCl, 2.2 CaCl_2_, 1.2 MgCl_2_, 14 glucose, and 10 HEPES; pH was adjusted to 7.4 with 10 N NaOH. Recording pipette solution used to measure the Cl_Ca_ current had the following composition: 120 CsCl, 20 tetraethylammonium-Cl, 2.8 MgCl_2_, 2 ATP-Na_2_, 10 HEPES, 5 EGTA, and 4.25 CaCl_2_; pH was adjusted to 7.2 with 1 N CsOH.

In the current-clamp mode, a series of depolarizing currents from 0 to 500 pA (150 ms) in 50 pA steps was used to trigger the action potential to measure the current threshold (rheobase) near the explosive action potential current. After that, another protocol step of 2 × rheobase (duration, 500 ms; amplitude, double intensity of 1 × rheobase) was performed to record the number of action potentials. For the current clamp experiments, the bath solution contained (in mM) 140 NaCl, 5 KCl, 2 CaCl_2_, 2 MgCl_2_, 10 D-glucose, and 10 HEPES; pH was adjusted to 7.4 with NaOH. The pipette solution contained (in mM) 30 KCl, 100 K-aspartate, 5 MgCl_2_, 2 Mg-ATP, 0.1 Na-GTP, and 40 HEPES; pH was adjusted to 7.2 with KOH.

### Quantification and Statistics

All data are expressed as the mean ± SEM and were analyzed using SPSS 16.0 software (SPSS Inc., Chicago, IL, USA). Statistical differences between two groups were analyzed by Student’s test. One-way analysis of variance followed by the Student–Newman–Keuls test was used to compare the differences between more than two groups. A *p*-value < 0.05 was considered statistically significant.

## Results

### TMEM16A Expression in DRG Neurons

**Fig. 1:**
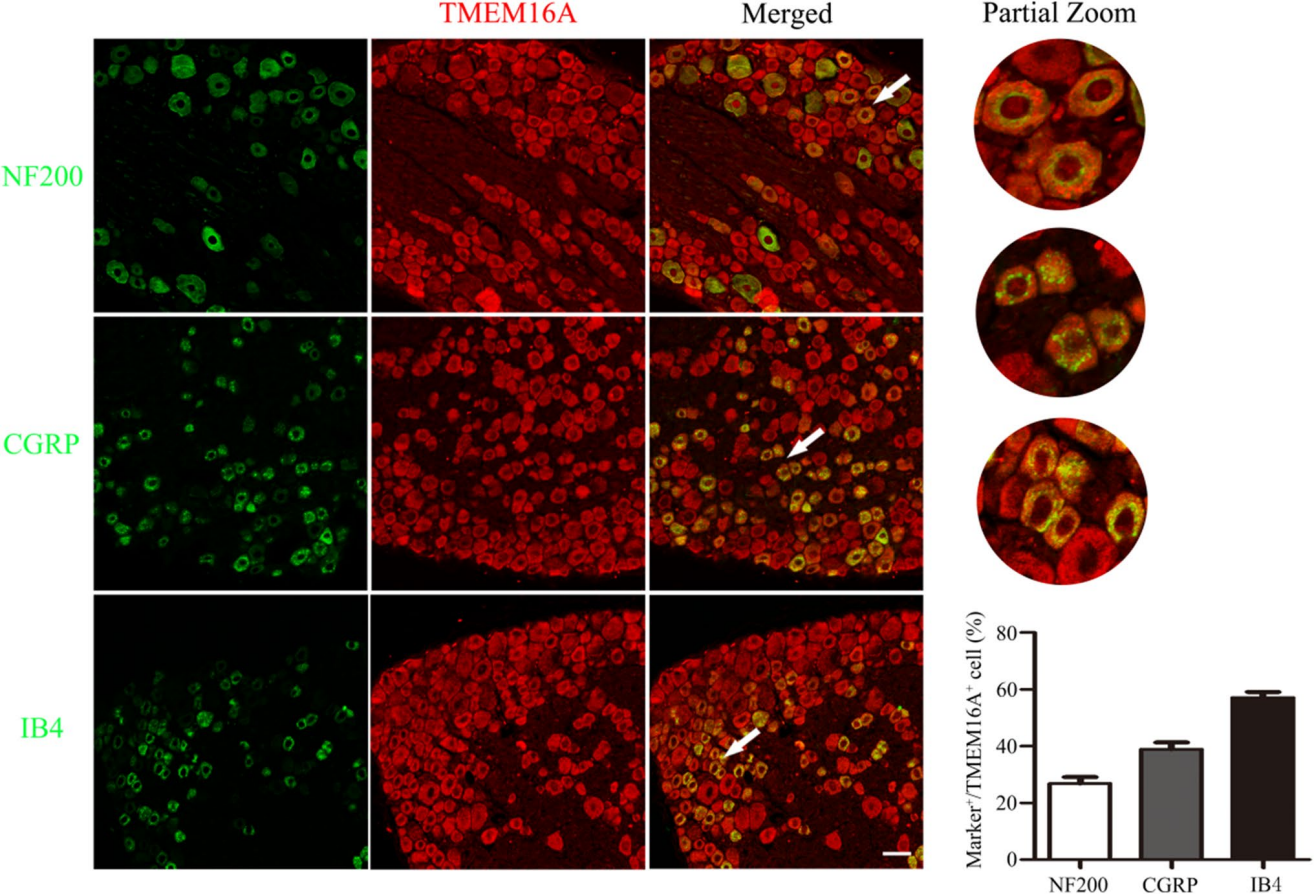
TMEM16A expression in DRG neurons. Double immunofluorescence staining showing that TMEM16A (red) is colocalized with IB4 (a marker of nonpeptidergic C-type neurons, green), CGRP (a marker of peptidergic C-type neurons, green) and NF-200 (a marker of A-type neurons, green). White arrows indicate the area of local magnification. Scale bar = 50 μm. Quantification of the coexpression of TMEM16A with IB4, CGRP and NF-200

To determine the function of TMEM16A in DRG neurons, we initially used immunofluorescence double staining to characterize the expression of TMEM16A in DRG neurons. The percentages of IB4 (a marker of nonpeptidergic C-type neurons), CGRP (a marker of peptidergic C-type neurons)-, and NF-200 (a marker of A-type neurons). Positive neurons relative to TMEM16A-positive cells were 57.1 ± 1.9%, 38.9 ± 2.4%, and 26.9 ± 2.2%, respectively (Fig. 1). These results demonstrate that TMEM16A has a higher expression ratio on C-type DRG neurons, indicating that TMEM16A may be involved in the regulation of superficial sensations, such as pain.

### SNI Rats Have Cold and Mechanical Allodynia and Are not Sensitive to Thermal Stimulation

**Fig. 2:**
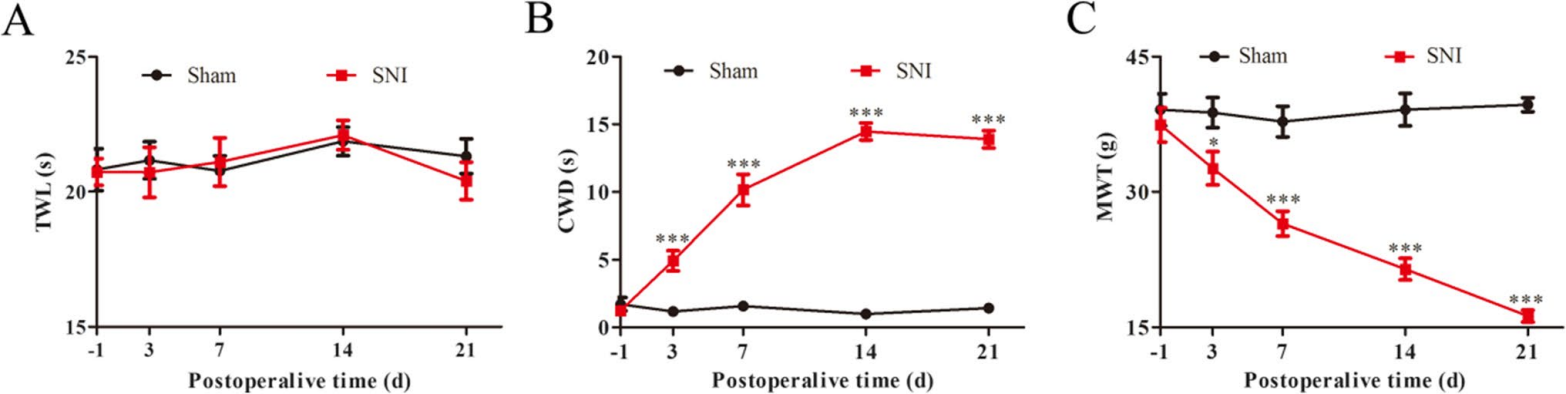
SNI rats are sensitive to cold and mechanical pain, but not to thermal stimulation. **A** Time courses of the withdrawal latency to thermal stimuli were determined in the sham and SNI groups. Radiant heat stimulus (IR 50) was applied to the left paws of the rats. **B** Time courses of the withdrawal duration to cold stimuli were measured in the sham and SNI groups. Acetone (100 μl) was used on the left hind paw of the rats. **C** Time courses of withdrawal thresholds in the sham and SNI groups to von Frey hair stimuli on day 1 before and on days 3, 7, 14, and 21 after the surgery. Mechanical stimuli were applied to the left hind paw of rats with von Frey hairs. *n* = 6, compared with the sham group at the same time point, *p < 0.05, **p < 0.01, ***p < 0.001

The results of statistical analysis of behavioral tests at various time points after the surgery (Fig. 2) showed that there are no statistically significant differences in the preoperative baseline values of thermal withdrawal latency (TWL), cold withdrawal duration (CWD), and mechanical withdrawal threshold (MWT) between the sham and SNI rats (*p* > 0.05). The TWL values in the SNI group were not statistically significantly different at each time point (*p* > 0.05) unlike those in the sham group (Fig. 2A). The CWD values in the SNI group were increased on day 3 after the operation (*p* < 0.001), reaching a peak on day 14 after the surgery (*p* < 0.001) and continuing to increase until day 21 after SNI (*p* < 0.001) (Fig. 2B). The MWT values in the SNI group were decreased on day 3 after the operation (*p* < 0.05) and continued to decrease until day 21 after SNI (*p* < 0.001) (Fig. 2C). The results indicate that SNI rats are not sensitive to thermal pain and have hyperalgesia to cold and mechanical stimuli.

### Changes in TMEM16A Expression on DRG After SNI

**Fig. 3:**
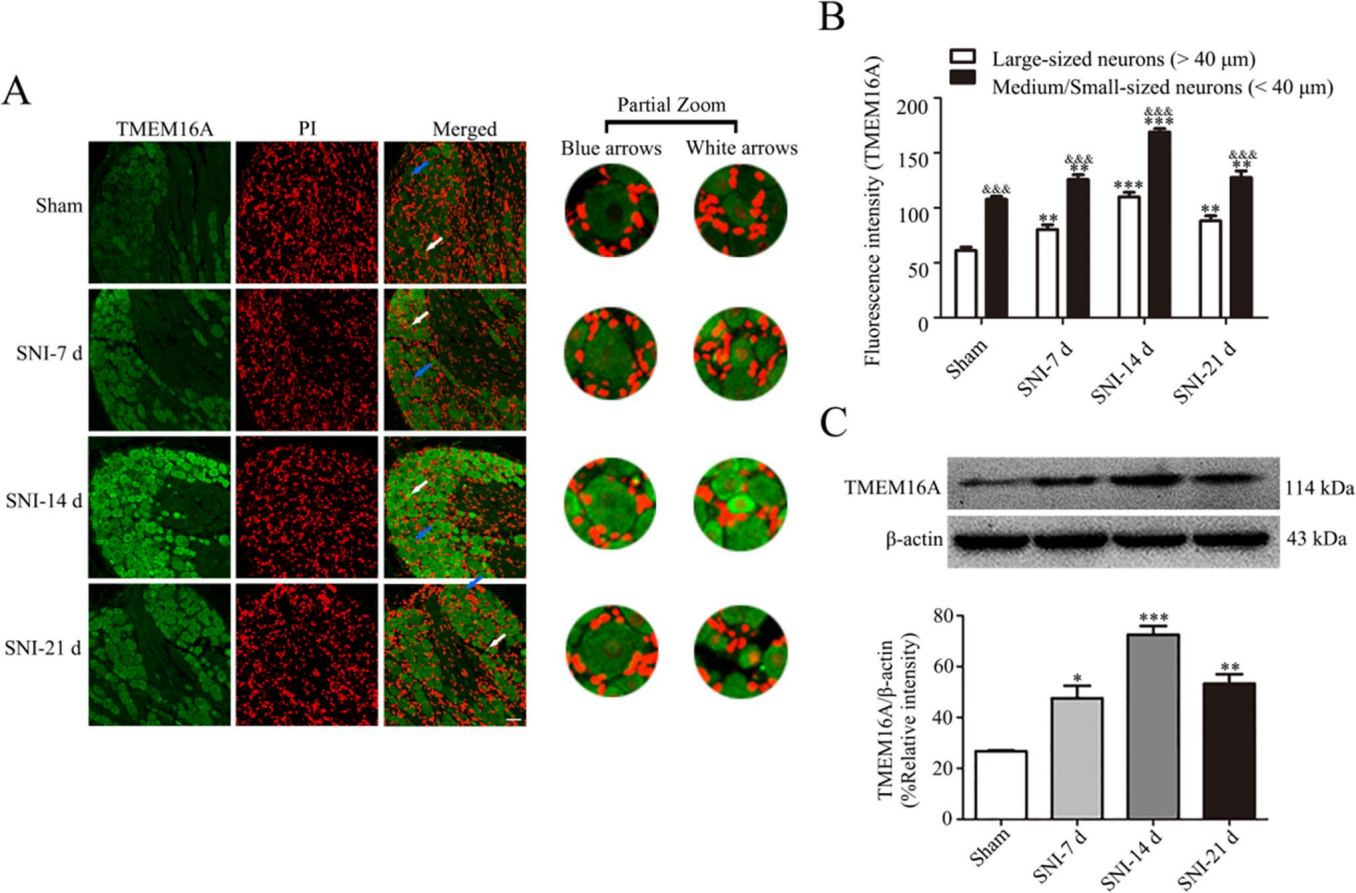
TMEM16A levels in DRG neurons are increased after SNI. **A** Immunofluorescence detection of the expression of TMEM16A based on fluorescence intensity in the dorsal root ganglia of rats in the SNI group at each time point. Blue arrows indicate representative large neurons; white arrows indicate representative medium-sized/small neurons. **B** The fluorescence intensity of TMEM16A in ipsilateral L4-L6 DRGs at various time points. Comparison with the sham group: *p < 0.05, **p < 0.01, ***p < 0.001 in two types of neuronal cells; comparison of medium-sized/small neurons with large neurons: ^&^*p* < 0.05, ^&&^*p* < 0.01, ^&&&^*p* < 0.001 in each group. Scale bar = 50 μm, *n* = 6. **C** Trends in the expression of TMEM16A in the dorsal root ganglia of SNI rats were detected by western blot at various time points. Comparison with the sham group: *p < 0.05, **p < 0.01, ***p < 0.001, *n* = 6

Immunofluorescence staining and western blotting of rat ipsilateral L4-L6 DRGs at various time points after spared nerve injury (SNI) showed that the protein levels of TMEM16A were higher than those in sham-operated rats on day 7 after the operation, and the upregulation of TMEM16A persisted for at least 21 days after SNI, reaching a maximum on day 14 (Fig. 3C). Notably, the TMEM16A fluorescence intensity in small and medium-sized neurons was higher than that in large neurons (*p* < 0.001) in each group (Fig. 3B). Thus, TMEM16A is expressed at a higher level in small and medium-sized neurons associated with pain signaling in DRG.

### The MEK/ERK Signaling Pathway Is Activated in DRG After SNI and Is Related to TMEM16A

**Fig. 4:**
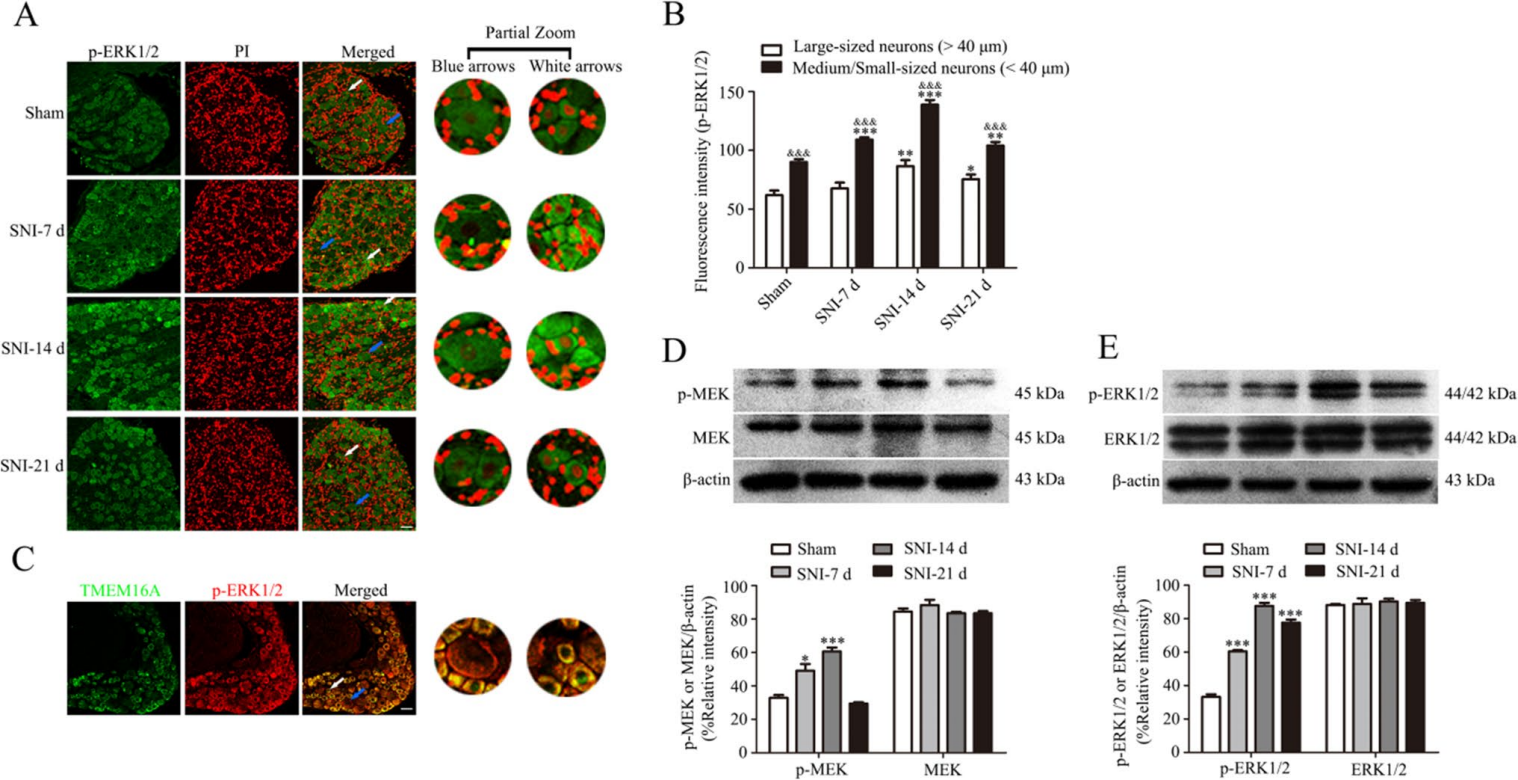
The MEK/ERK signaling pathway is activated in DRG after SNI, and p-ERK1/2 and TMEM16A are coexpressed at high levels. **A** Immunofluorescence was used to detect the expression based on fluorescence intensity in the dorsal root ganglia of rats at various time points. Blue arrows indicate representative large neurons; white arrows indicate representative medium-sized/small neurons. **B** The fluorescence intensity of the p-ERK1/2 signal in ipsilateral L4-L6 DRGs at various time points. Comparison with the sham group: ^*^p < 0.05, ^**^p < 0.01, ^***^p < 0.001 in two types of neuronal cells; medium-sized/small neurons were compared with large neurons: ^&^*p* < 0.05, ^&&^*p* < 0.01, ^&&&^*p* < 0.001 in each group. Scale bar = 50 μm, *n* = 6. C. Immunofluorescence coexpression of TMEM16A and p-ERK1/2 in the dorsal root ganglia of rats on day 14 after SNI. Scale bars = 50 μm, *n* = 6. **D**–**E** The protein expression of p-MEK, MEK (D) and p-ERK1/2, ERK1/2 (E) in the dorsal root ganglia of SNI rats at each time point, *n* = 6; comparison with the sham group: ^*^p < 0.05, ^**^p < 0.01, ^***^p < 0.001

Immunofluorescence assays were performed in the dorsal root ganglia of SNI rats at various time points (Fig. 4A). After SNI, the fluorescence intensity of the p-ERK1/2 signal was increased in small, medium-sized, or large neurons (*p* < 0.05) and peaked on day 14 after the surgery (*p* < 0.001). In all groups, the fluorescence intensity of the *p*-ERK1/2 signal in small and medium-sized neurons was higher than that in large neurons (*p* < 0.001) (Fig. 4B) suggesting that p-ERK1/2 is expressed mainly in pain-related small and medium-sized neurons in the dorsal root ganglia.

Western blot was used to assay the expression levels of the MEK/ERK signaling components, including phosphorylated MEK (Ser221) and ERK1/2 (Thr202/Tyr204); the results indicated that the levels were significantly increased on day 7 after SNI compared with those in the sham group, reaching a maximum on day 14 (Fig. 4D, E). Thus, the MEK/ERK signaling pathway is activated after SNI.

The results of double immunofluorescence staining revealed that TMEM16A is always predominantly colocalized with p-ERK1/2 in L4-L6 DRGs of SNI rats on postoperative day 14 (Fig. 4C). These results suggest that activation of the MEK/ERK signaling pathway may be associated with TMEM16A after SNI.

### Increased NK-1 Expression Is Related to p-ERK1/2 Activation in DRG After SNI

**Fig. 5:**
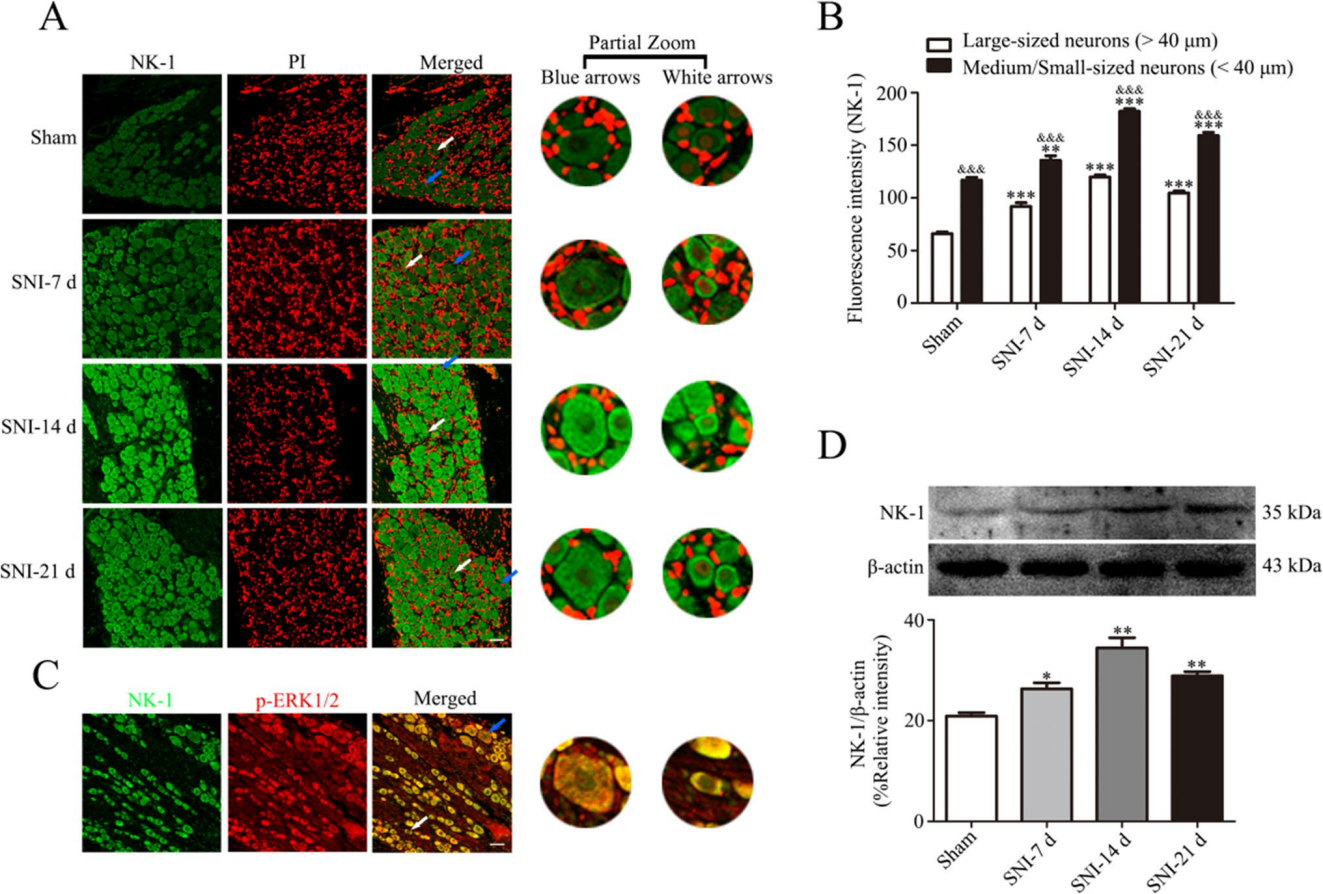
NK-1 expression increased after SNI and co-expressed with p-ERK1/2 on DRG. **A** Immunofluorescence was used to detect the expression of NK-1 based on the fluorescence intensity of the signal in the dorsal root ganglia of rats in the SNI group at all time points. Blue arrows indicate representative large neurons; white arrows indicate representative medium-sized/small neurons. **B** The fluorescence intensity of NK-1 signal in ipsilateral L4-L6 DRGs at various time points. Comparison with the sham group: ^*^p < 0.05, ^**^p < 0.01, ^***^p < 0.001 in two types of neuronal cells; medium-sized/small neurons were compared with large neurons: ^&^*p* < 0.05, ^&&^*p* < 0.01, ^&&&^*p* < 0.001 in all groups. Scale bar = 50 μm, *n* = 6. **C** Immunofluorescence assay of the coexpression of NK-1 and p-ERK1/2 in the dorsal root ganglia of rats on day 14 after SNI. Scale bars = 50 μm, *n* = 6. **D** The expression of NK-1 in the dorsal root ganglia of rats after SNI at all time points, *n* = 6; comparison with the sham group: ^*^p < 0.05, ^**^p < 0.01, ^***^p < 0.001

The results of the immunofluorescence and western blot assays suggested that NK-1 is mainly expressed in small and medium-sized neurons associated with pain; the protein expression was the highest on day 14 after SNI (Fig. 5B, D). Double immunofluorescence staining showed that NK-1 and p-ERK1/2 are colocalized in the dorsal root ganglia (Fig. 5C) suggesting that an increase in the expression of NK-1 may be associated with ERK1/2 phosphorylation after SNI.

### MEK Inhibitor U0126 Prevents Cold and Mechanical Allodynia Induced by SNI and Decreases the Expression of NK-1

**Fig. 6:**
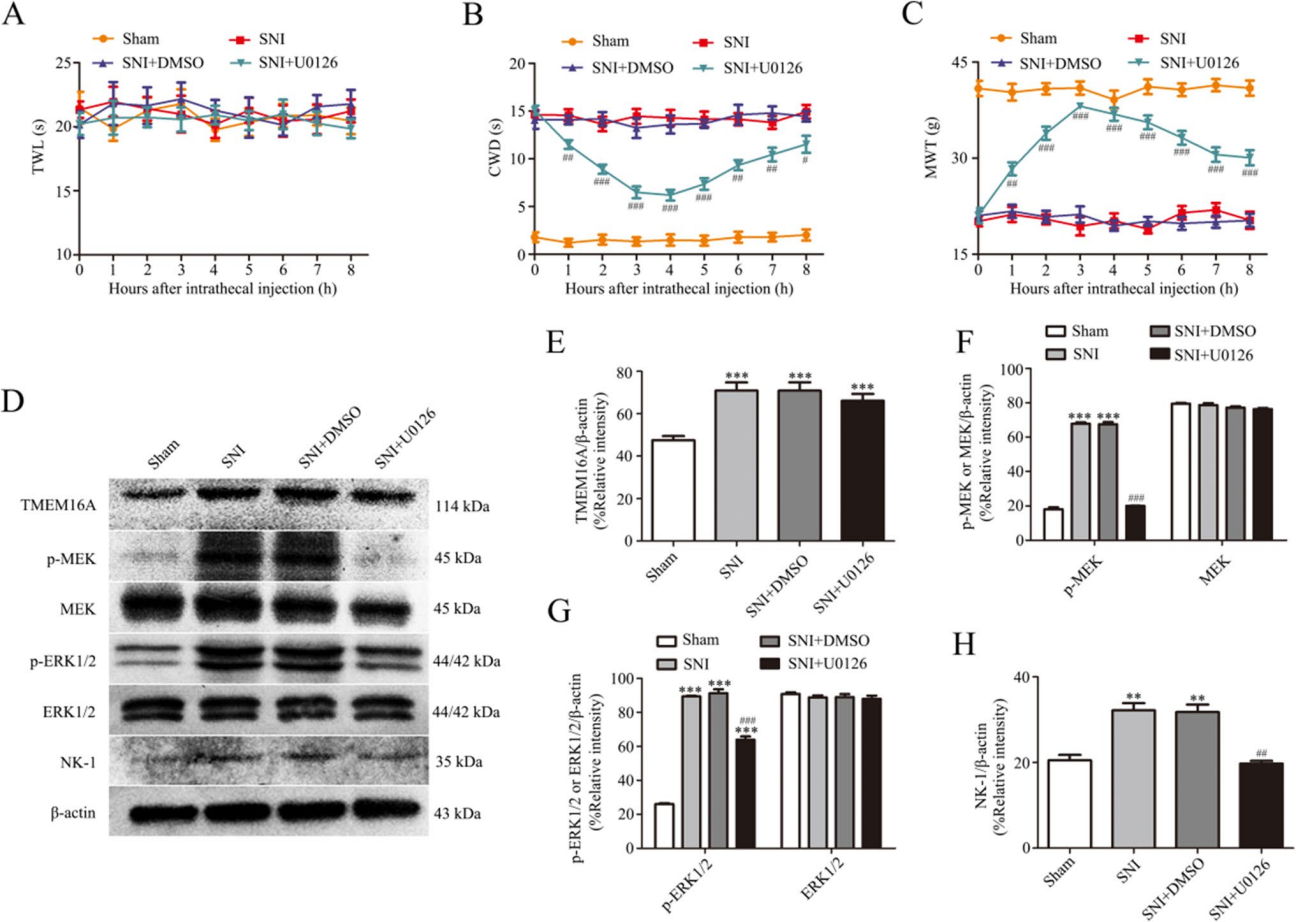
MEK inhibitor U0126 inhibits the development of cold and mechanical allodynia and increases the expression of NK-1 in L4-L6 DRGs after SNI. **A**–**C** Effect of MEK inhibitor U0126 on TWL, CWD, and MWT in SNI rats. Behavioral changes in TWL (A), CWD (B), and MWT (C) in four groups of rats at all time points. Comparison with the SNI + DMSO group: ^#^*p* < 0.05, ^##^*p* < 0.01, ^###^*p* < 0.001, *n* = 6. **D**–**H** Effect of MEK inhibitor U0126 on TMEM16A (E), p-MEK, MEK (F), p-ERK1/2, ERK1/2 (G), and NK-1 (H) expression in the spinal dorsal root ganglia of rats after SNI. Comparison with the sham group: ^*^p < 0.05, ^**^p < 0.01, ^***^p < 0.001; comparison with the SNI + DMSO group: ^#^*p* < 0.05, ^##^*p* < 0.01, ^###^*p* < 0.001, *n* = 6

To determine the role of the MEK/ERK signaling pathway in pain and its relationship with NK-1, we investigated the changes in pain behavior and NK-1 expression in rats after intrathecal administration of U0126. The results showed that a MEK-specific inhibitor, U0126, significantly alleviated cold and mechanical allodynia in rats after SNI (Fig. 6A–C, p < 0.05). The animal model of SNI is not sensitive to thermal stimulation; hence, there was no significant change in thermal pain in SNI rats after intrathecal administration of U0126.

The results of western blot assay indicated that the expression levels of p-MEK, p-ERK1/2, and NK-1 in the SNI group treated with U0126 were significantly reduced compared to those in the DMSO-treated SNI rats (Fig. 6F–H, p < 0.01). However, U0126 did not have any effect on the expression of TMEM16A (Fig. 6E), indicating that MEK inhibitor U0126 can downregulate the expression of NK-1 in L4-L6 DRGs of rats after SNI but does not influence the expression of TMEM16A after SNI.

### TMEM16A Antagonist T16Ainh-A01 Prevents SNI-Induced Cold and Mechanical Allodynia and ERK Phosphorylation and Increases NK-1 in L4-L6 DRGs

**Fig. 7:**
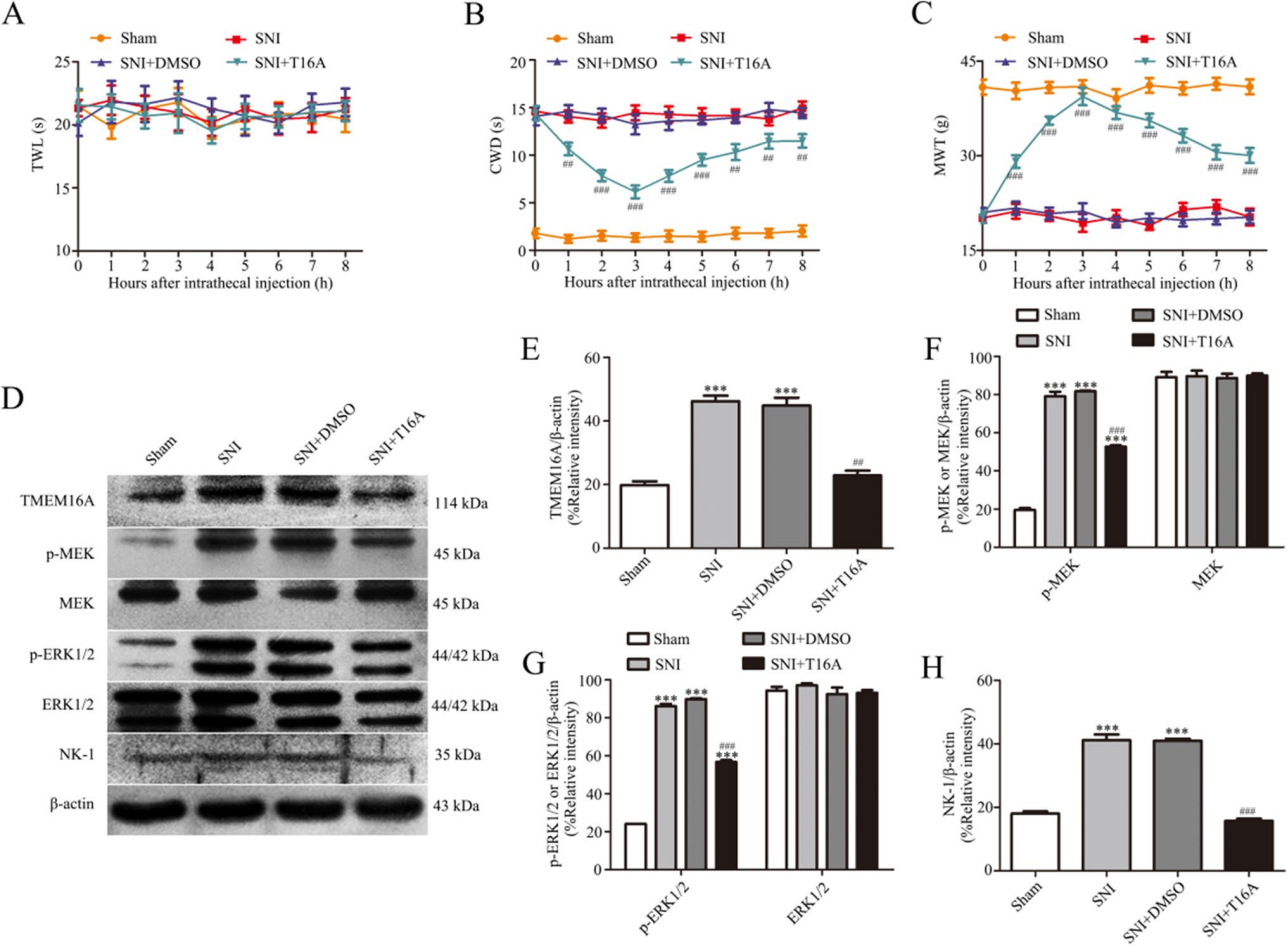
TMEM16A antagonist T16Ainh-A01 relieves cold and mechanical allodynia and reduces ERK phosphorylation and NK-1 expression after SNI. **A**–**C** Effect of TMEM16A inhibitor T16Ainh-A01 on TWL, CWD, and MWT in rats after SNI. Behavioral changes in TWL (A), CWD (B), and MWT (C) of four groups of rats at all time points. Comparison with the SNI + DMSO group: ^#^*p* < 0.05, ^##^*p* < 0.01, ^###^*p* < 0.001, *n* = 6. **D**–**H** Effect of TMEM16A inhibitor T16Ainh-A01 on TMEM16A (E), p-MEK, MEK (F), p-ERK1/2, ERK1/2 (G), and NK-1 (H) expression in the spinal dorsal root ganglia of rats after SNI. Comparison with the sham group: ^*^p < 0.05, ^**^p < 0.01, ^***^p < 0.001; comparison with the SNI + DMSO group: ^#^*p* < 0.05, ^##^*p* < 0.01, ^###^*p* < 0.001, *n* = 6

To determine the role of TMEM16A in SNI-induced allodynia, a selective inhibitor of TMEM16A, T16Ainh-A01 (10 µg in 10 µl), was intrathecally injected 5 min before behavioral tests. The antihyperalgesic effects were evaluated during the following 8 h. Behavioral tests showed that T16Ainh-A01, but not DMSO, blocks the development of cold and mechanical allodynia after SNI (Fig. 7A–C, p < 0.01).

To determine whether phosphorylation of ERK and upregulation of NK-1 are mediated by TMEM16A, we investigated the effect of TMEM16A antagonist T16Ainh-A01 on the induction of p-ERK and NK-1 in L4-L6 DRGs. The results of the western blot assay indicated that the expression levels of TMEM16A, p-MEK, p-ERK1/2, and NK-1 in T16Ainh-A01-treated SNI rats were significantly reduced compared to those in DMSO-treated rats after SNI (Fig. 7E–H, p < 0.01). The data of the western blot assay confirmed that T16Ainh-A01 inhibits the activation of the MEK/ERK signaling pathway and downregulates the expression of NK-1 in L4-L6 DRGs after SNI.

### CaCC Current Increases After SNI

**Fig. 8:**
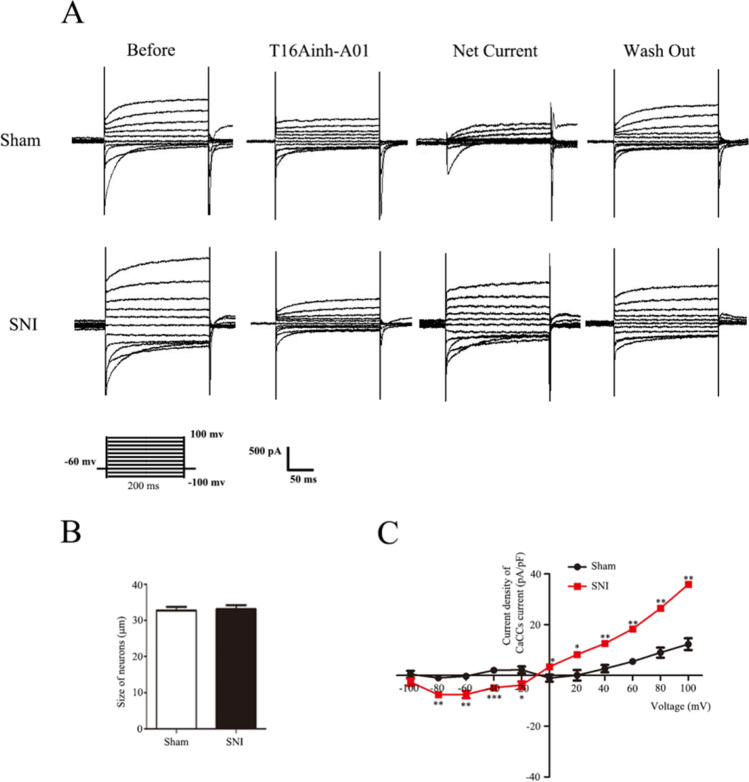
Changes in the current density of CaCCs after using T16Ainh-A01 in the dorsal root ganglion of rats after SNI. **A** Representative tracings showing the current in DRG neurons after the step protocol (from -100 to + 100 mV in 20 mV increments with a pulse duration of 100 ms). Before: tracing before T16Ainh-A01 was used. T16Ainh-A01: tracings after T16Ainh-A01 incubation for 60 s. Net current: tracings obtained by subtracting the tracings of “T16Ainh-A01” from the tracings of “Before.” Wash out: tracing after the extracellular fluid was washed for 4 min. **B** Histogram showing the size of neurons. **C** Current density–voltage curves of DRG neurons in the sham and SNI groups. Comparison with the sham group: ^*^p < 0.05, ^**^p < 0.01, ^***^p < 0.001; *n* = 6

To investigate whether the CaCC current has changed in DRG neurons after SNI, patch-clamp experiments were performed. Chloride current was measured in rat DRG neurons under the whole-cell patch-clamp conditions by using K^+^-deficient and Cl^−^-enriched solutions. Single DRG neurons were depolarized from a holding potential of 0 mV to a selected test potential (-100 to + 100 mV) in 1000 ms increments of 20 mV. T16Ainh-A01 (20 μM) can specifically block CaCC currents, and CaCC currents can be restored after washing DRG cells with extracellular fluid for 30 s. The “Net” current was obtained by subtracting the T16Ainh-A01 from the “Before” current, which represents the current sensitive to T16Ainh-A01, and the Net current was used to represent the current carried by CaCC (Fig. 8A). For a more accurate measure of the current change, the current density was calculated. All DRG neurons were harvested on the 14th day after surgery. The DRG for patch clamp was incubated with T16Ainh-A01 in vitro. In addition, the size of all neurons was between 20 and 35 µm (Fig. 8B).

At the voltage of − 100 mV, there was no significant statistical difference in the current density between the sham group and the SNI group. Starting from the -80mv voltage, compared with the sham group, the current density of the SNI group began to increase, and the increase was more and more significant. The current–voltage relationship indicates that the reversal potential is 0 mV.

### T16Ainh-A01 Decreased the Excitability of DRG Neurons Caused by SNI

**Fig. 9:**
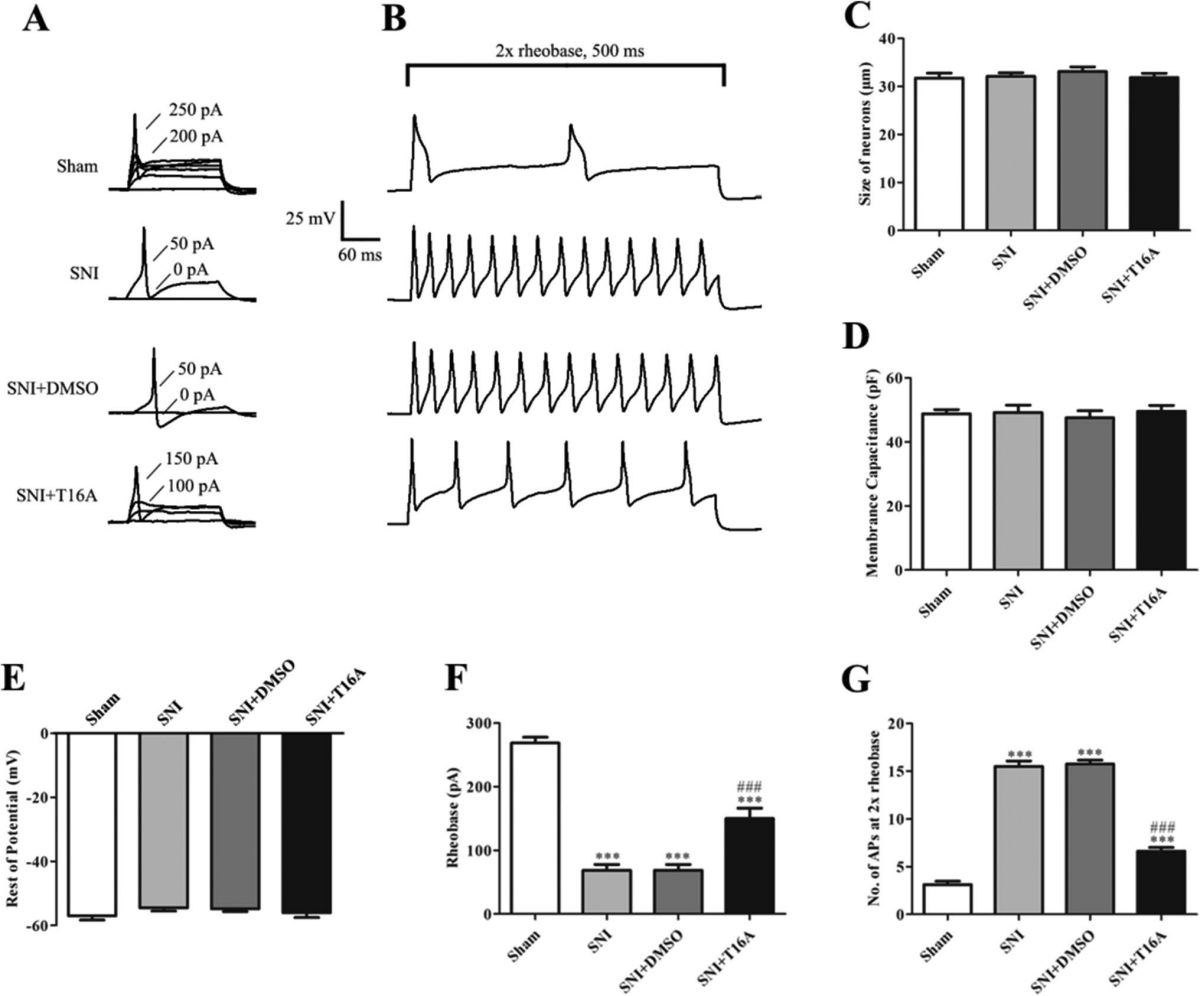
T16Ainh-A01 reduces enhanced DRG excitability in rats after SNI. **A** Representative tracings of rheobases of action potentials evoked by current injections in DRG neurons of rats in all groups. **B** Typical tracings showing the action potentials elicited by doubled intensity of rheobase for 500 ms in DRG neurons of rats in all groups. **C** Histogram showing the size of neurons. **D** Histogram showing the membrane capacitance. **E** Histogram showing the resting potential. **F** Histogram showing the statistical comparison of rheobase of the action potentials in each group. **G** Histogram showing the statistical comparison of the number of action potentials elicited by doubled rheobase intensity for 500 ms in all groups. Comparison with the sham group: *p < 0.05, **p < 0.01, ***p < 0.001; comparison with the SNI + DMSO group: ^#^ *p* < 0.05, ^##^ *p* < 0.01, ^###^ *p* < 0.001

To examine why T16Ainh-A01 reduced cold and mechanical allodynia in SNI rats, we explored the characteristics of DRG neuron action potentials. In the current clamp mode, the action potentials were triggered by a series of depolarizing currents from 0 to 500 pA (150 ms) in increments of 50 pA to measure the current threshold (rheobase), i.e., the minimal current that evoked an action potential, which was used as an indicator of DRG neuron excitability (Fig. 9). All DRG neurons were harvested on the 14th day after surgery. The DRG for patch clamp was incubated with T16Ainh-A01 in vitro.

As shown in Fig. 9, 1 × rheobase of the action potential was significantly reduced in the SNI group (68.75 ± 10.32 pA, *p* < 0.001) compared with that in the sham group (268.75 ± 9.24 pA). The number of action potentials was significantly increased in the 2 × rheobase group (15.50 ± 1.63, *p* < 0.001) compared with that in the sham group (3.13 ± 1.52). The application of T16Ainh-A01 (TMEM16A blocker) increased 1 × rheobase of the action potential (150.0 ± 25.82 pA, *p* < 0.001) and significantly reduced the number of action potentials in the 2 × rheobase mode (6.63 ± 0.82, *p* < 0.001) compared with those in the SNI + DMSO group (68.75 ± 8.23 pA, 15.75 ± 1.32). There were no significant differences between the SNI and SNI + DMSO groups in 1 × rheobase value or the number of action potentials in the 2 × rheobase mode. There were no significant differences in other action potential parameters among the groups, such as membrane capacitance and resting membrane potential (Fig. 9D, E). In addition, the size of all neurons was between 20 and 35 µm (Fig. 9C).

### Intrathecal Injection of TMEM16A Agonist E-act Produces Allodynia and Increases p-MEK, p-ERK, and NK-1 Expression in L4-L6 DRGs

**Fig. 10:**
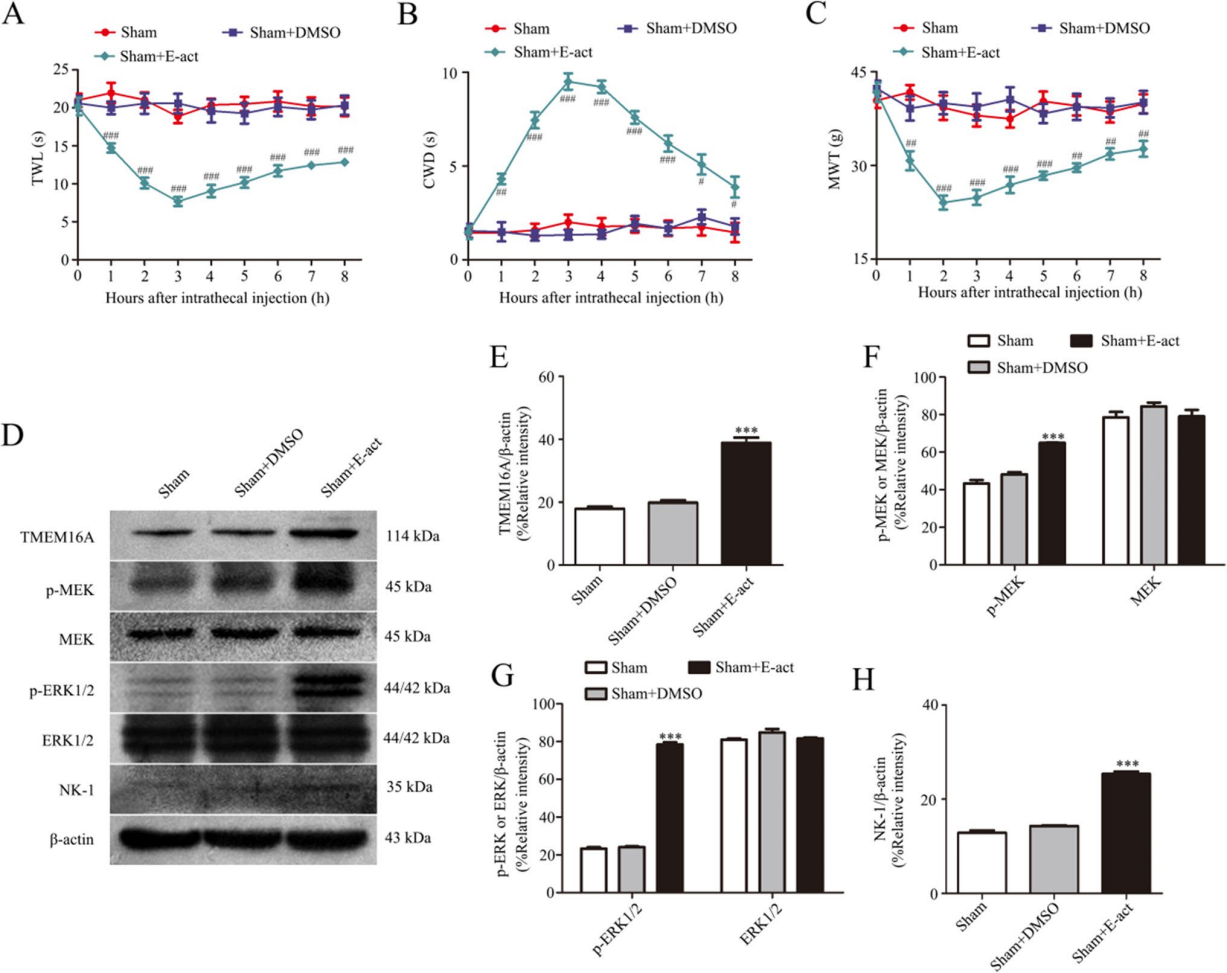
Intrathecal injection of TMEM16A agonist E-act induces thermal, cold, and mechanical allodynia and p-MEK, p-ERK, and NK-1 expression in L4-L6 DRGs. **A**–**C** Effect of TMEM16A agonist E-act on TWL, CWD, and MWT in sham rats. Behavioral changes in TWL (A), CWD (B), and MWT (C) in three groups of rats at all time points. **D**–**H** Effect of TMEM16A agonist E-act on TMEM16A (E), p-MEK, MEK (F), p-ERK1/2, ERK1/2 (G), and NK-1 (H) expression in the spinal dorsal root ganglia of sham rats. Comparison with the sham group: ^*^p < 0.05, ^**^p < 0.01, ^***^p < 0.001; comparison with the SNI + DMSO group: ^#^*p* < 0.05, ^##^*p* < 0.01, ^###^*p* < 0.001, *n* = 6

The behavioral test showed that the cold withdrawal duration in the bilateral hind paw was increased, and the thermal withdrawal latency and the mechanical withdrawal threshold were decreased following intrathecal injection of TMEM16A agonist E-act (10 μg in 10 μl) 5 min before evaluation (Fig. 10A–C), indicating that E-act produces bilateral thermal hyperalgesia and cold and mechanical allodynia. Intrathecal injection of DMSO did not influence the pain threshold. The administration of E-act to promote the activation of TMEM16A significantly influences the basic pain threshold in rats.

Western blot results confirmed that the TMEM16A agonist E-act activates the MEK/ERK signaling pathway and upregulates the expression of NK-1 in L4-L6 DRGs of sham rats (Fig. 10E–H).

### TMEM16A Agonist E-act Boosts DRG Excitability

To investigate the effect of TMEM16A activation on DRG excitability, treatment with a TMEM16A agonist, E-act, was used to detect the changes in rheobase and number of the action potentials. All DRG neurons were harvested on the 14th day after surgery. The DRG for patch clamp was incubated with E-act in vitro.

**Fig. 11:**
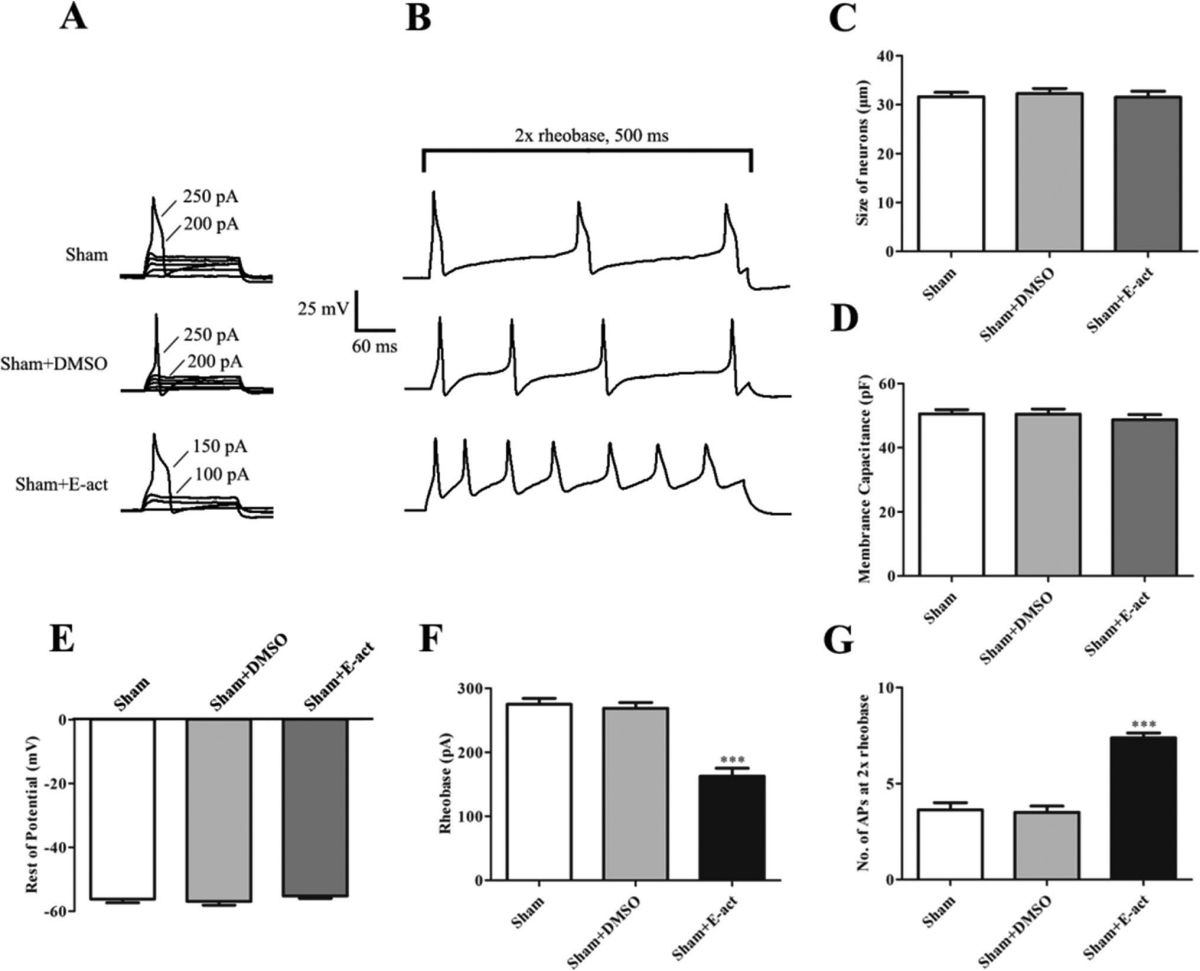
E-act enhances DRGs excitability in L4-L6 DRG. **A** Representative tracings of rheobases of action potentials evoked by current injections in DRG neurons of rats in all groups. **B** Typical tracings of the action potentials elicited by doubled rheobase intensity for 500 ms in DRG neurons of rats in all groups. **C** Histogram showing the size of neurons. **D** Histogram showing the membrane capacitance. **E** Histogram showing the resting potential. **F** Histogram showing the statistical comparison of rheobase of the action potentials in all groups. **G** Histogram showing the statistical comparison of the number of action potentials elicited by doubled rheobase intensity for 500 ms in all groups. Comparison with the sham group: ^*^p < 0.05, ^**^p < 0.01, ^***^p < 0.001

As shown in Fig. 11, 1 × rheobase was significantly reduced (100 ± 12.91 pA, *p* < 0.001) compared with that in the sham group (366.67 ± 10.54 pA) (Fig. 11F), and the number of the action potentials was significantly increased (18.83 ± 0.60, *p* < 0.001) (Fig. 11G) in the 2 × rheobase mode after administration of E-act. There were no significant differences in the rheobase value or number of the action potentials in the 2 × rheobase mode between the sham and sham + DMSO groups. There were no significant differences in other action potential parameters among the groups, such as membrane capacitance and resting membrane potential (Fig. 11D, E). In addition, the size of all neurons was between 20–35 µm (Fig. 11C).

## Discussion

Nociceptive impulses originate from primary afferent neurons in the dorsal root or trigeminal ganglion and activate neurons in the spinal cord and specific nuclei in the brain to produce pain. Sensory transduction in DRG neurons is achieved by activating a specific class of ion channels [28]. These channels are molecular sensors that detect harmless and harmful stimuli and convert them into electrical pulses.

TMEM16A is expressed in DRG neurons [10]; hence, it is reasonable to suggest that TMEM16A is involved in somatosensory transduction. Our data indicate that TMEM16A is mainly expressed in small and medium-sized neurons associated with pain. DRG neurons of various sizes have different responses to various stimuli [29]. Small and medium-sized neurons respond specifically to harmful thermal, chemical, and mechanical stimuli and are therefore considered multimodal nociceptors. Behavioral tests showed CWD reached its highest value on the 14th day, while MWT was the 21st day. It showed that the onset of cold allodynia was faster than the onset of mechanical allodynia, and cold allodynia reached its peak at an earlier time than mechanical allodynia in SNI animals. The TMEM16A protein also expressed the most on the 14th day, indicating that TMEM16A may be more closely related to cold allodynia in SNI rats. It is reported that the SNI animals maintained strong mechanical allodynia throughout the entire observation period of 85 days [30]. TMEM16A has a downward trend on the 21st day, indicating that other proteins may be involved in subsequent mechanical pain in SNI rats.

Recent studies on DRG suggest that TMEM16A and TRPV1 are colocalized, indicating that they play a role in nociception [31]. Moreover, TMEM16A can be activated by temperature above 44 °C [31]. Knockout of TMEM16A in DRG neurons causes a significant loss of heat pain [31]. These results indicate that TMEM16A apparently acts as a sensor that mediates or amplifies thermal nociception. Interestingly, sham rats treated with TMEM16A-specific agonist E-act showed a behavioral pain of thermal hyperalgesia; however, TMEM16A was activated after nerve damage, and SNI rats were not sensitive to heat pain. This result indicates that other mechanisms influence the perception of thermal stimulation in rats after SNI and regulate heat pain; however, these specific mechanisms are unclear.

Numerous studies have shown that ERK signaling pathways play an important role in pain regulation [14, 32, 33]. ATP activation of TMEM16A is mediated by an increase in intracellular Ca^2+^, and a Ca^2+^-independent mechanism is associated with ERK1/2 [34]. Previous studies demonstrated that overexpression of TMEM16A promotes the proliferation of squamous cell carcinoma of the head and neck accompanied by activation of the ERK1/2 pathway [23]. Similarly, elevated TMEM16A expression in the breast cancer and ovarian granulosa cells can induce ERK1/2 phosphorylation, while knockdown of TMEM16A or pharmacological inhibition of the channel activity reduces ERK1/2 activation [21, 35]. These data suggest that ERK1/2 activity is related to TMEM16A activation. Additionally, Duvvuri demonstrated that overexpression of TMEM16A activates the MEK/ERK pathway but does not induce phosphorylation of Akt and ERK5 [23]. These results suggest that TMEM16A activates the ERK1/2 pathway. Therefore, our study investigated the relationship between TMEM16A and the MEK/ERK pathways in dorsal root ganglia.

ERK1/2 can regulate the expression of NK-1. NK-1 is a G protein-coupled receptor that can influence multiple signaling pathways in the cells, such as two signaling pathways related to IP3 and DAG [36]. DAG opens the L-type calcium channel in the plasma membrane through protein kinase C, and IP3 acts on specific receptors in the sarcoplasmic reticulum to release Ca^2+^ stores in the cells to significantly increase the intracellular Ca^2+^ concentration, promote the release of transmitters or inflammatory factors, and influence the transcriptional regulation of the genes to trigger the subsequent activation of a broader range of the downstream signaling pathways. NK-1 activation can induce thermal hyperalgesia via PKCε-mediated TRPV1 enhancement [37]. Bee venom reduces mechanical allodynia of chronic postischaemic pain (CPIP) by reducing NK-1 expression [38]. Therefore, NK-1 processing is essential for excitatory transmission and nociceptive information [39]. NK-1 mRNA has an untranslated cAMP response element-binding protein (CREB) binding site at the 5′-end unlike two other receptors, and the MEK/ERK signaling pathway can influence the transcription factor CREB by phosphorylating ERK1/2 in the nucleus, thereby regulating the expression of the pain factor NK-1 to produce pain [20]. The results of the present study indicate that TMEM16A is activated in the dorsal root ganglia after SNI; additionally, the MEK/ERK signaling pathway is activated, and NK-1 expression is increased. To define the upstream and downstream relationships between these three factors, we administered TMEM16A and MEK inhibitors and observed the behavioral changes in rats after SNI and changes in protein expression in the dorsal root ganglia.

**Fig. 12:**
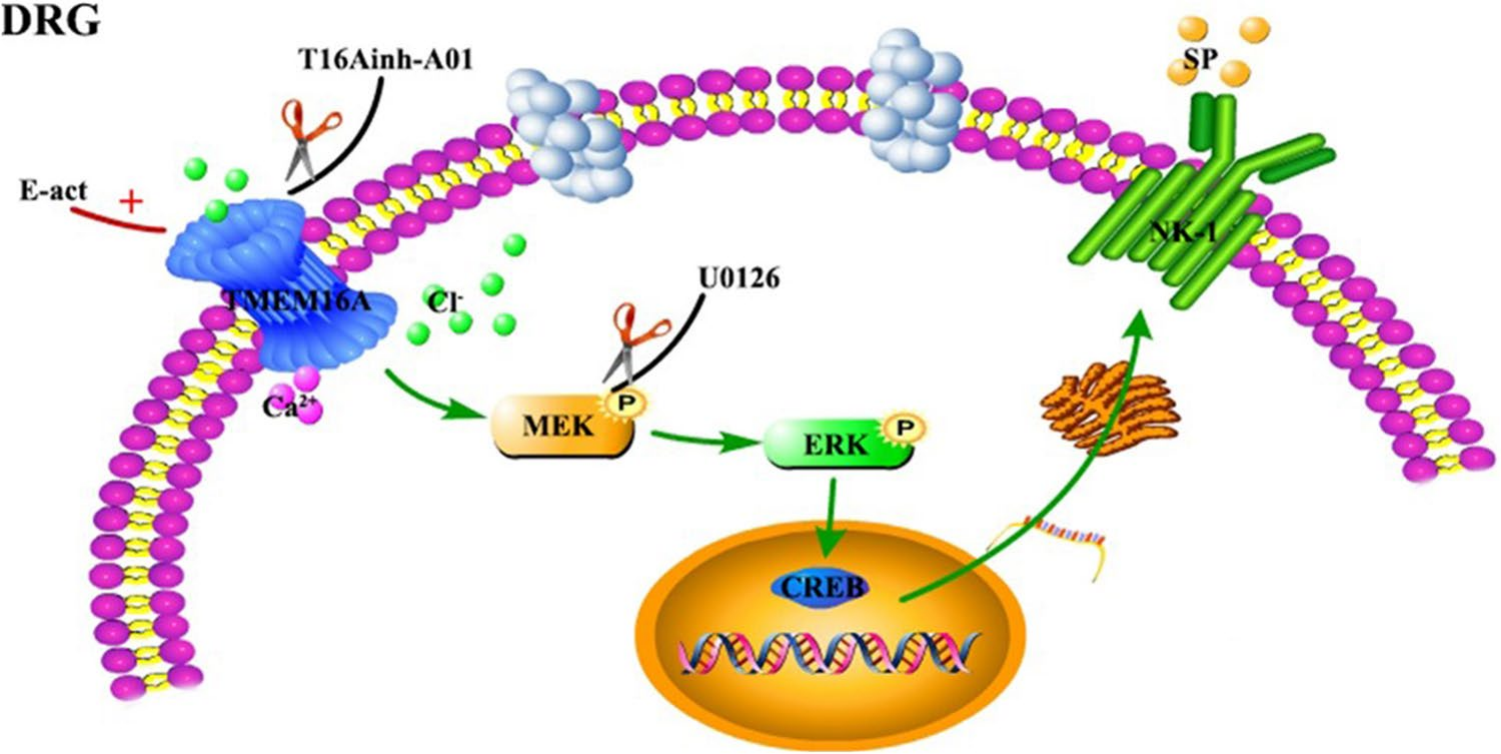
The signaling pathways activated by TMEM16A in SNI. Schematic diagram of the mechanism of the TMEM16A-mediated regulation of neuropathic pain in rats after SNI. TMEM16A may influence the expression of the substance P receptor NK-1 through the MEK/ERK signaling pathway, thereby participating in neuropathic pain. However, this effect can be blocked by T16Ainh-A01 or U0126; E-act can activate TMEM16A and cause pain. T16Ainh-A01 is a specific blocker of TMEM16A; U0126 is a specific blocker of MEK, and E-act is an agonist of TMEM16A

This study is the first to use behavioral tests and immunofluorescence and molecular biology techniques to determine the role and status of TMEM16A in DRG in neuropathic pain in spared nerve injury (SNI) that is a neuropathic pain model. The results indicate that the expression level of TMEM16A in the dorsal root ganglia of rats after SNI is significantly increased, the MEK/ERK signaling pathway is activated, and the expression of NK-1 is significantly increased. The results of immunofluorescence staining showed that the levels of TMEM16A, p-ERK1/2, and NK-1 were increased in neurons related to pain after SNI. Thus, these three factors may play an important role in the regulation of nociceptive information. Importantly, intrathecal injection of a specific inhibitor of TMEM16A, T16Ainh-A01, significantly alleviates cold and mechanical allodynia induced by SNI and simultaneously inhibits the expression of p-MEK and p-ERK1/2 phosphorylation in the MEK/ERK signaling pathway and downregulates the expression level of NK-1. Intrathecal administration of MEK inhibitor U0126 significantly reduces cold and mechanical hyperalgesia after SNI and inhibits the overexpression of NK-1 but does not influence the expression of TMEM16A. Intrathecal injection of TMEM16A-specific agonist E-act in sham rats induces hyperalgesia and upregulates the expression of p-MEK, p-ERK1/2, and NK-1 (Fig 12). These results indicate that TMEM16A in rat spinal dorsal root ganglion neurons can mediate NK-1 overexpression by activating the MEK/ERK signaling pathway and participates in the transformation of chronic neuropathic pain after SNI.

Ga^2+^ is an endogenous ligand of TMEM16A that can significantly enhance the sensitivity of TMEM16A to heat pain. An increase in intracellular Ca^2+^ concentration enhances the TMEM16A current induced by thermal stimulation and lowers the temperature threshold of TMEM16A activation below 44 ℃ [31]. For example, an increase in intracellular Ca^2+^ above 0.5 μM activates TMEM16A at a temperature close to the body temperature of 37.5 ℃. Nerve damage can increase intracellular Ca^2+^ [40],thus, TMEM16A can be sensitized only by normal body temperature under these pathological conditions. The regulation of intracellular Cl^−^ concentration in sensory neurons was suggested to be involved in the regulation of hyperalgesia or transmission of nociceptive signals [41, 42]. TMEM16A blocker T16Ainh-A01 can influence the function of TMEM16A to change the intracellular Cl^−^ concentration and thus influence pain behavior. Nerve injury-induced nociceptive behavior is significantly attenuated by TMEM16A blocker T16Ainh-A01 [43]. If the concentration of Cl^−^ in the neurons increases after nerve injury, TMEM16A activation enhances the driving force of Cl^−^ for depolarization. Previous studies in our laboratory demonstrated that nerve injury increases the intracellular Cl^−^ concentration and enhances the excitability of the dorsal root ganglion to promote allodynia [3] thus contributing to the intermediate role of TMEM16A in chronic pain.

TMEM16A is a Cl^−^ channel; thus, opening of TMEM16A can lead to hyperpolarization or depolarization of neurons in an intracellular chloride concentration ([Cl^−^]_i_)-dependent manner. [Cl^−^]_i_ in DRG neurons is regulated primarily by sodium–potassium-chloride cotransporter 1 (NKCC1), which accumulates Cl^−^ in the cells [42]. Thus, unlike neurons in the CNS, DRG neurons maintain higher [Cl^−^]_i_ levels than those present in electrochemical equilibrium because of elevated NKCC1 expression and activity [44]. The equilibrium potential of Cl^−^ is considerably more positive (–27 mV) than the resting membrane potential (–60 to − 55 mV) in DRG neurons [44–46],thus, the activation of Cl^−^ channels in DRG neurons leads to depolarization. However, we repeatedly observed that sensory neuronal TMEM16A has outwardly rectifying current–voltage curves similar to the current–voltage curves recorded for native TMEM16A in other tissues [47, 48]. This phenomenon implies that TMEM16A carries relatively low currents associated with Cl^−^ efflux. It is possible that currents of other channels, voltage regulation of TMEM16A, [Ca^2+^]_i_ modulation of TMEM16A currents, or other factors mask TMEM16A-dependent inward currents (Cl^−^ efflux) in the sensory neuronal current–voltage curves. TMEM16A outward rectifying currents imply that TMEM16A carries a large influx of Cl^−^ once APs are activated. Thus, TMEM16A can enhance the repolarization phase of APs at depolarizing potentials.

Previous studies have reported that spinal nerve ligation can increase spinal dorsal root ganglia excitability, which is partly caused by CaCC activation [43]. T16Ainh-A01, a specific inhibitor of TMEM16A, can partially reduce an increase in dorsal root ganglion excitability caused by spinal cord ligation [43]. Intrathecal injection of nonselective and selective TMEM16A inhibitors (T16Ainh-A01) [43] or TMEM16A knockdown [13] can reduce mechanical allodynia and thermal hyperalgesia caused by nerve damage. Intradermal injection of TMEM16A-specific agonist E-act in naïve mice can produce pruritus, acute nociception, and thermal hypersensitivity [49]. The results of our study indicated that after nerve injury, TMEM16A expression is significantly increased, CaCC currents are increased, and DRG excitability is increased. TMEM16A-specific inhibitor T16Ainh-A01 can inhibit this increase and hyperalgesia caused by SNI. The mechanism of activation of CaCCs by nerve injury is unclear. However, it is known that nerve damage can cause peripheral and central sensitization, which leads to activation of a large number of excitatory mechanisms and increases intracellular Ca^2+^ levels [50]. This response is sufficient to activate CaCCs, cause chloride ion efflux, generate inward currents that depolarize the membrane [15, 16], and eventually cause abnormal pain after SNI.

We suggest that during SNI, nerve damage induces an increase in intracellular Ca^2+^ to cause TMEM16A activation in DRG neurons. On the one hand, TMEM16A is activated as an ion channel that induces efflux of Cl^−^ resulting in depolarization of the membrane and certain effects on neuronal excitability. On the other hand, TMEM16A can mediate the activation of the MEK/ERK signaling pathway to increase NK-1 expression, leading to enhanced axon-dependent NK-1 transport to the periphery or central end, causing peripheral sensitization and final production of neuropathic pain. In summary, this study demonstrates that inhibition of TMEM16A in DRG neurons reduces hyperalgesia caused by nerve damage. Thus, TMEM16A in the spinal dorsal root ganglion neurons can be used as a new therapeutic target for the prevention of neuropathic pain.

## Supplementary Information

Below is the link to the electronic supplementary material.

Supplementary file1 (DOCX 379 KB)

Supplementary file2 (DOCX 1574 KB)

## Data Availability

The datasets generated for this study are available on request to the corresponding author.
