# Supplementary material for: The Role of TMEM16A/ERK/NK-1 Signaling in Dorsal Root Ganglia Neurons in the Development of Neuropathic Pain Induced by Spared Nerve Injury (SNI)
Source: Mol Neurobiol. 2021 Aug 18;58(11):5772–89. doi: 10.1007/s12035-021-02520-9 (PMC8599235; doi:10.1007/s12035-021-02520-9)
Supplement: Supplementary file 1 — Supplementary file1 (DOCX 379 KB) [file 12035_2021_2520_MOESM1_ESM.docx]

Supplementary Materials

**1. Experiment protocol**

**1.1. Experiment 1**

To determine the role of TMEM16A in DRG, colocalization of TMEM16A with specific neuronal markers was detected by immunofluorescence in DRG neurons of naïve rats. Then, to determine the behavioral pain characteristics of the rats and alterations of TMEM16A, the MEK/ERK signaling pathway and NK-1 expression in neuropathic pain caused by SNI, SD rats were randomly divided into the SNI group and sham operation group. Behavioral tests were performed at the following five time points: day 1 before SNI surgery (baseline), day 3 after SNI, day 7 after SNI, day 14 after SNI and day 21 after SNI. All rats were euthanized after behavioral tests at each time point, and ipsilateral L4-L6 DRGs were harvested to evaluate the expression of TMEM16A, the MEK/ERK signaling pathway and NK-1 by immunofluorescence and western blotting. Additionally, on day 14 after SNI, colocalization of TMEM16A with p-ERK and p-ERK with NK-1 in DRG neurons was assayed by immunofluorescence.

**1.2. Experiment 2**

To investigate the mechanism of action of TMEM16A in neuropathic pain in SNI rats, SD rats were randomly divided into four groups: the sham group, SNI group, SNI+5%DMSO group and SNI+T16Ainh-A01 (T16A) or U0126 group. T16Ainh-A01 is a specific inhibitor of TMEM16A, and U0126 is a specific inhibitor of MEK. T16Ainh-A01 and U0126 were delivered through an intrathecal catheter on day 14 after SNI operation. Behavioral tests were performed every 1 h within 8 h after administration.

Since SNI increased the expression of TMEM16A and the MEK/ERK signaling pathway, we detected the changes in the expression levels of TMEM16A, p-MEK, p-ERK1/2 and NK-1 in the presence and in the absence of TMEM16A or U0126 inhibitors after intrathecal administration of T16Ainh-A01 (10 μg) or U0126 (10 μg) every 6 h five times starting from day 12 after the surgery. DRG samples were obtained on day 14 after nerve injury.

To investigate whether the antiallodynic effect of TMEM16A inhibitor is mediated by a reduction in peripheral nerve injury-induced hyperexcitability, the CaCC current and action potential were recorded in L4-L6 DRGs of sham and SNI rats in the presence and in the absence of TMEM16A inhibitor T16Ainh-A01 (20 μM).

**1.3. Experiment 3**

To confirm the relationships of TMEM16A, the MEK/ERK signaling pathway and NK-1, SNI rats were randomly divided into 3 groups: the sham group, sham+5%DMSO (DMSO) group, and sham+E-act group. E-act is a specific agonist of TMEM16A. E-act was delivered through an intrathecal catheter on day 14 after the operation. Behavioral tests were performed every 1 h within 8 h after administration. E-act was intrathecally administered (10 μg) every 6 h five times starting from day 12 after the surgery. DRG samples were obtained on day 14 after the sham operation.

To investigate whether the allodynic effect of TMEM16A agonist was mediated by peripheral nerve hyperexcitability, the action potential was recorded in L4-L6 DRGs of sham rats in the presence and in the absence of TMEM16A agonist E-act (10 μM).


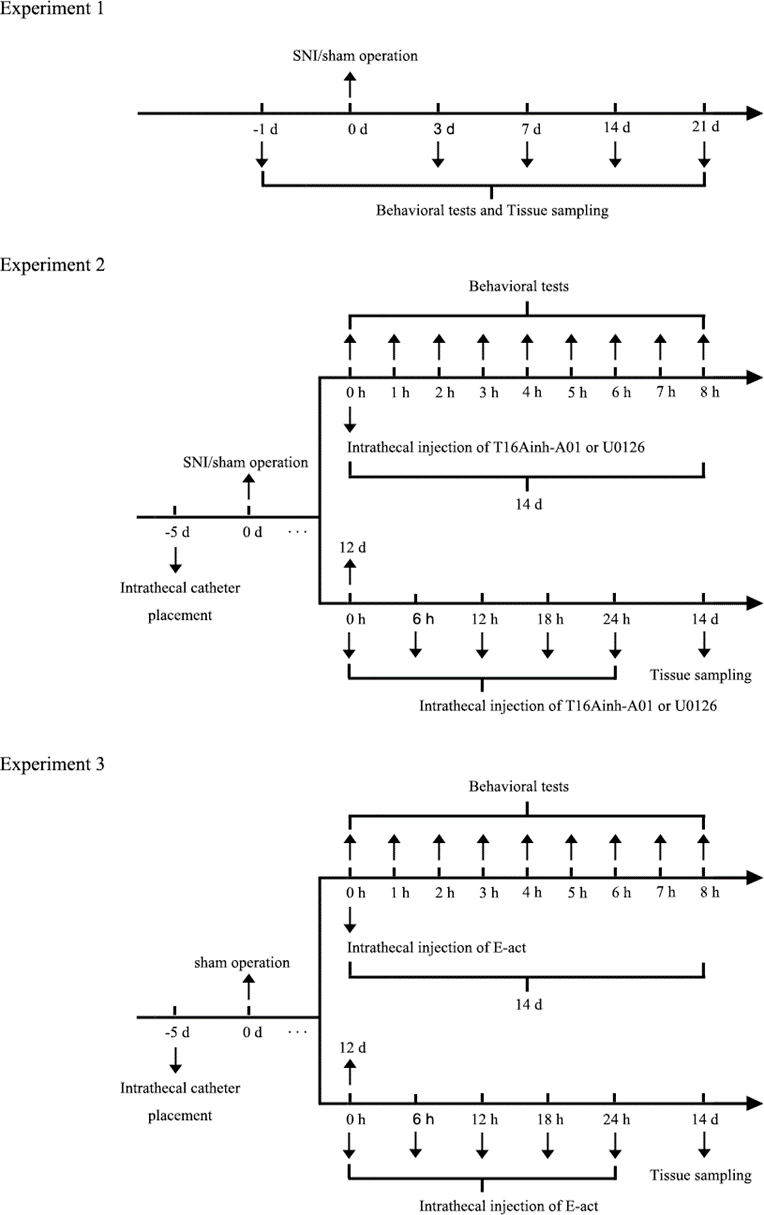


Fig. 1. Illustration of the experimental design.

Experiment 1: Protocol used to investigate the dynamic alterations of rat pain behaviors and TMEM16A, the MEK/ERK signaling pathway and NK-1 expression in DRG in a neuropathic pain model induced by SNI. Experiment 2: Protocol used to investigate the effect of intrathecal injection of T16Ainh-A01 or U0126 on pain behaviors and the expression of TMEM16A, the MEK/ERK signaling pathway and NK-1 in rats after SNI. Experiment 3: Protocol used to confirm the correlation of TMEM16A, the MEK/ERK signaling pathway and NK-1 via intrathecal injection of E-act.

**2. T16A and U0126 have no obvious effect on the pain behavior of Sham rats**

The figures below are the statistical graphs of the effects of T16Ainh-A01 and U0126 on the pain behavior (Fig. 2) and action potential (Fig.3) of sham-operated rats. Through statistical analysis, there is no significant statistical difference between the data in each group. The results indicated that T16Ainh-A01 and U0126 would not affect the pain behavior and action potentials in sham-operated rats.


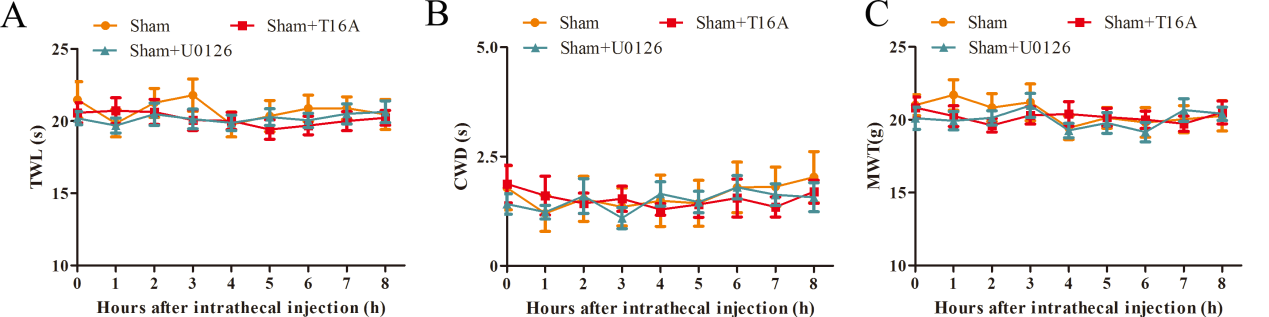


Fig. 2. The effect of T16A and U0126 on pain behavior in Sham rats

Effect of TMEM16A inhibitor T16Ainh-A01 (T16A) and MEK inhibitor U0126 on TWL (A), CWD (B) and MWT (C) in Sham rats. Comparison with the Sham group: ^*^p < 0.05, n=6.


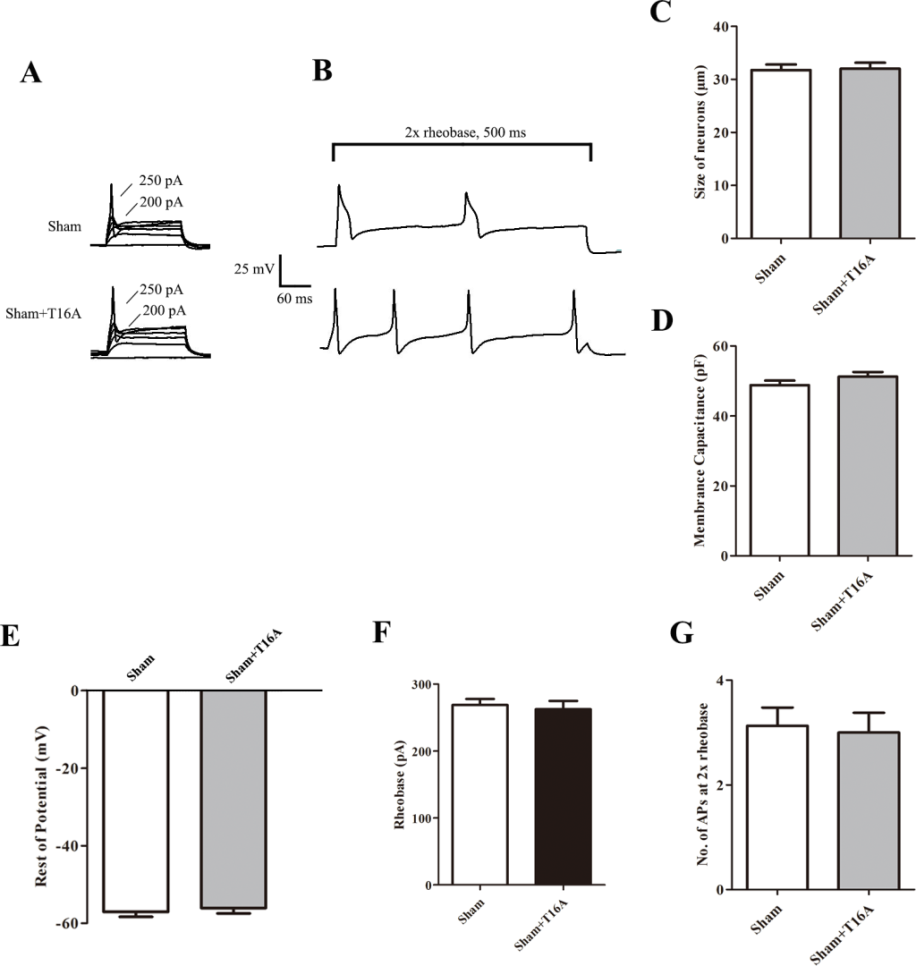


Fig. 3. The effect of T16Ainh-A01 on the excitability of DRG in Sham rats.

A. Representative tracings of rheobases of action potentials evoked by current injections in DRG neurons of rats in both groups. B. Typical tracings showing the action potentials elicited by doubled intensity of rheobase for 500 msec in DRG neurons of rats in both groups. C. Histogram showing the size of neurons. D. Histogram showing the membrane capacitance. E. Histogram showing the resting potential. F. Histogram showing the statistical comparison of rheobase of the action potentials in each group. G. Histogram showing the statistical comparison of the number of action potentials elicited by doubled rheobase intensity for 500 msec in all groups. Comparison with the sham group: *p <0.05.
