# Supplementary figures and images for: The Role of TMEM16A/ERK/NK-1 Signaling in Dorsal Root Ganglia Neurons in the Development of Neuropathic Pain Induced by Spared Nerve Injury (SNI)
Source: Mol Neurobiol. 2021 Aug 18;58(11):5772–89. doi: 10.1007/s12035-021-02520-9 (PMC8599235; doi:10.1007/s12035-021-02520-9)

**The original full-film image of western blot**

**Figrue 3**


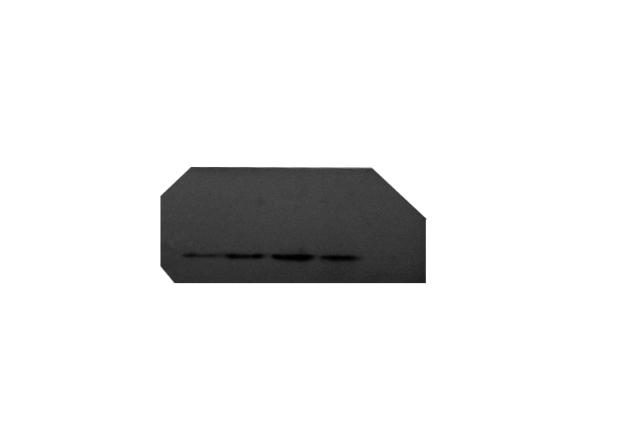

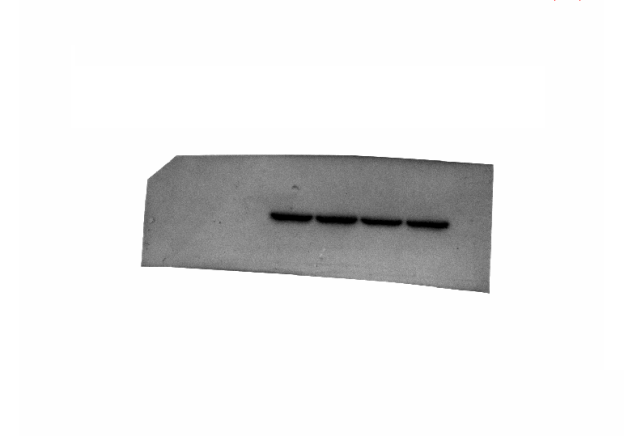


**Figure 4**


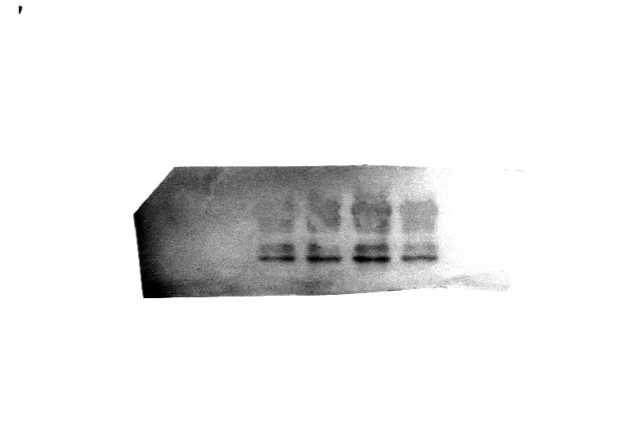

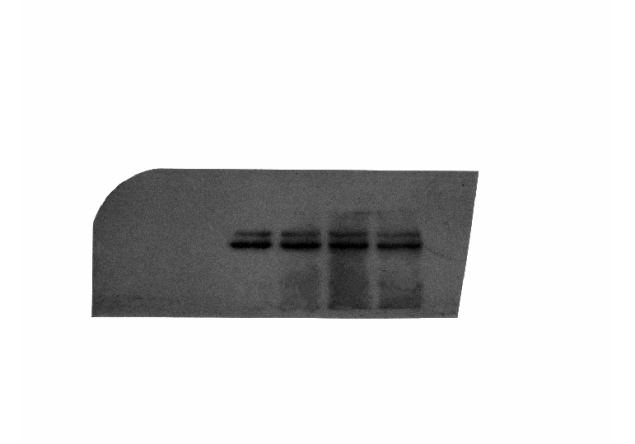


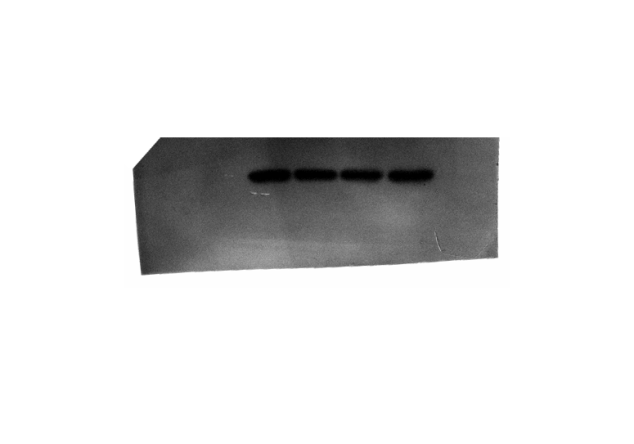

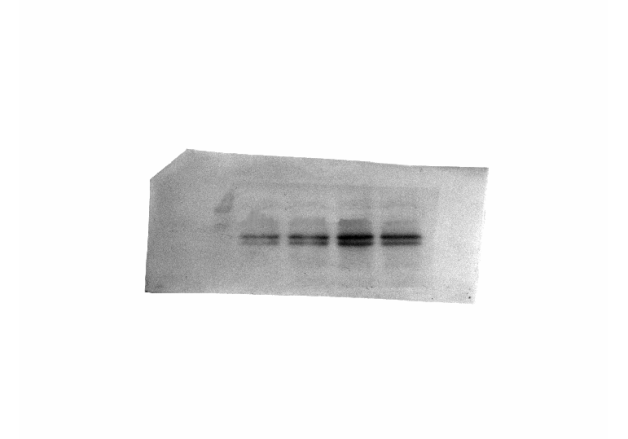


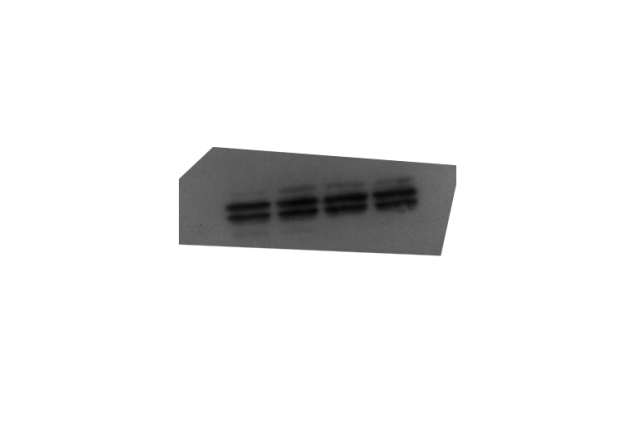

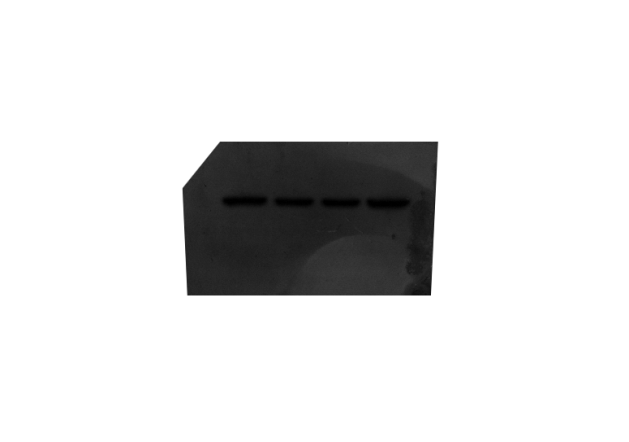


**Figure 5**


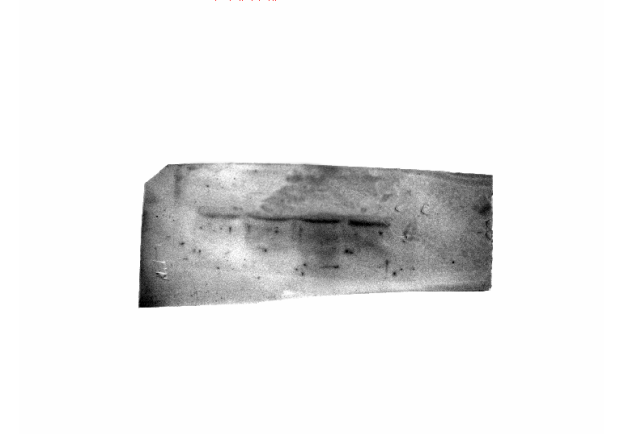

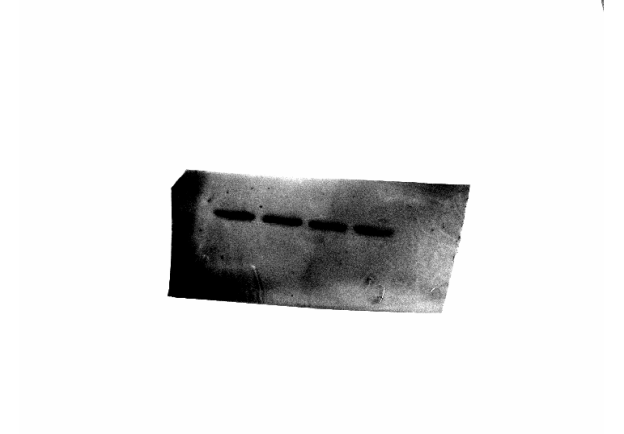


**Figure 6**


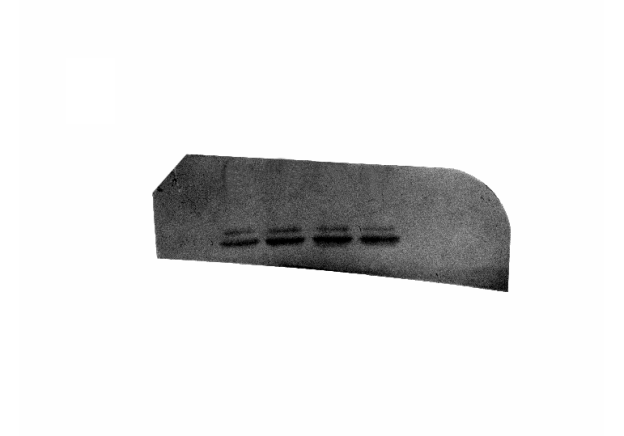

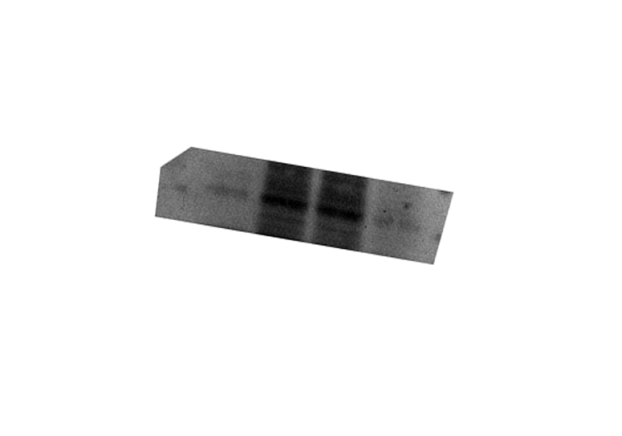


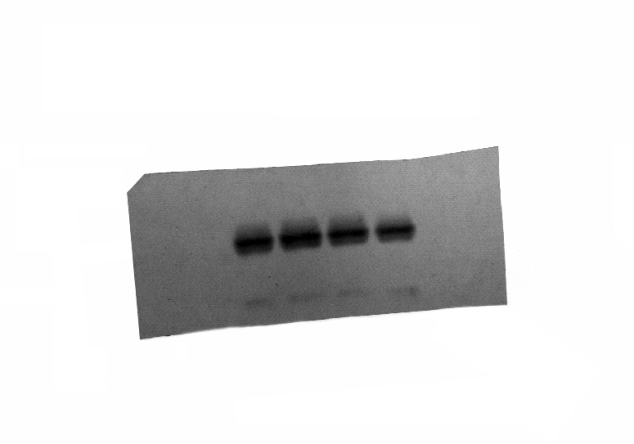

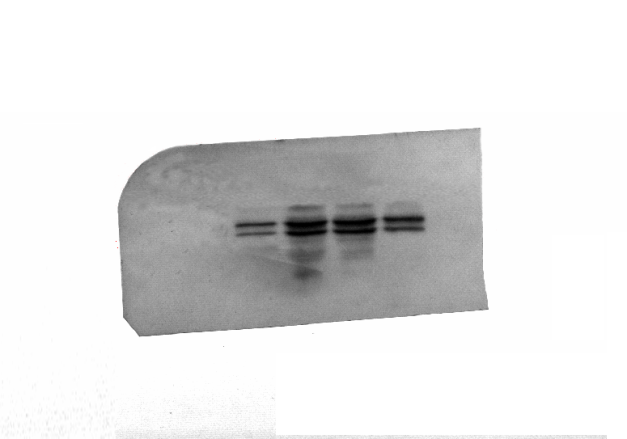


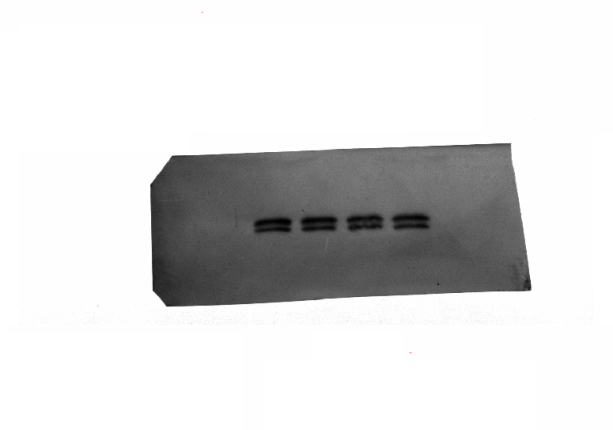

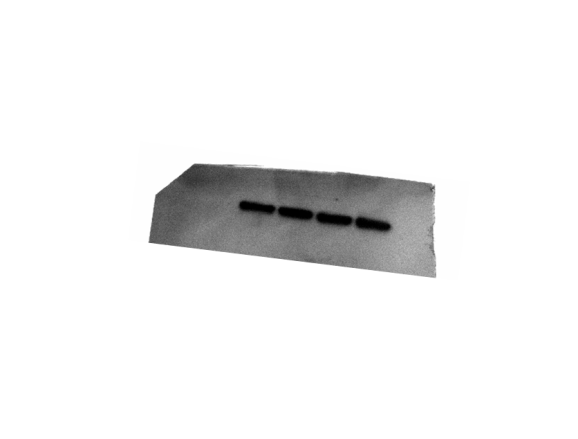


**Figure 7**


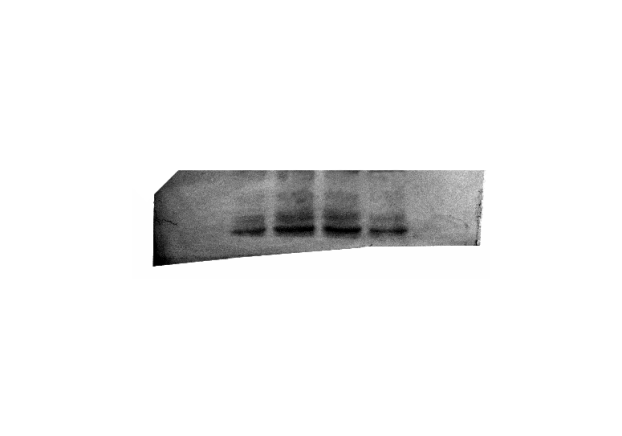

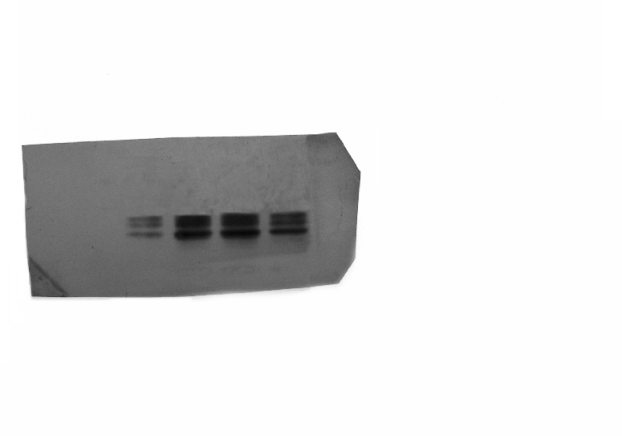


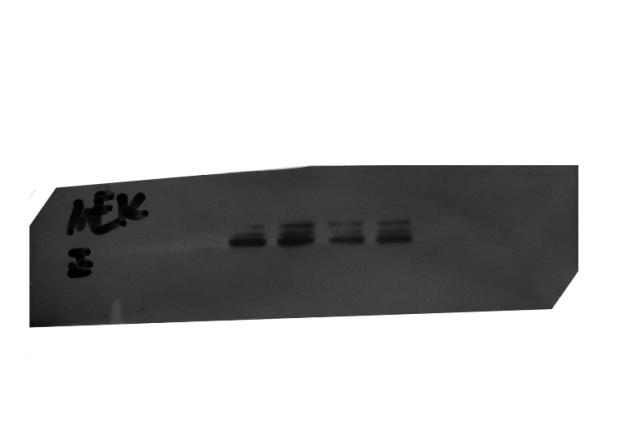

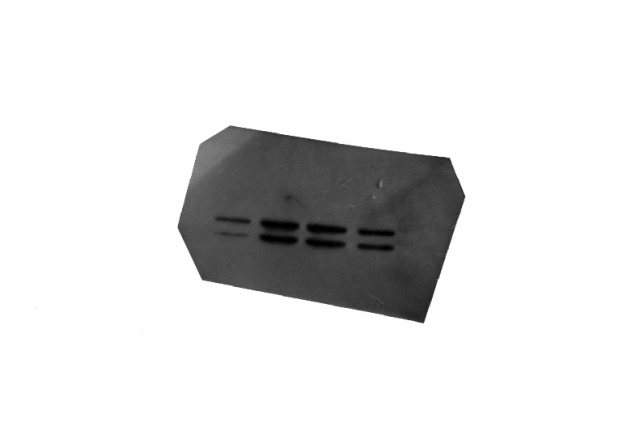


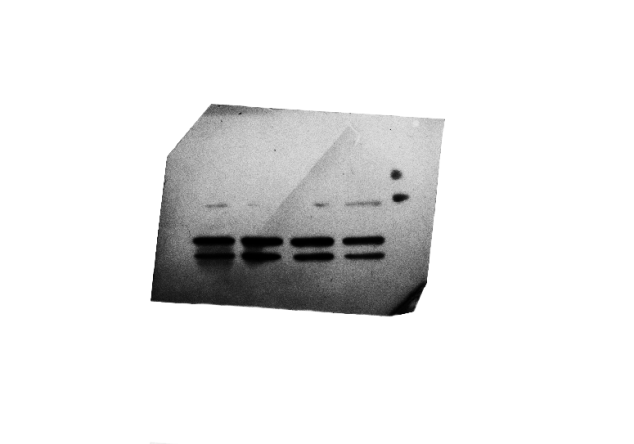

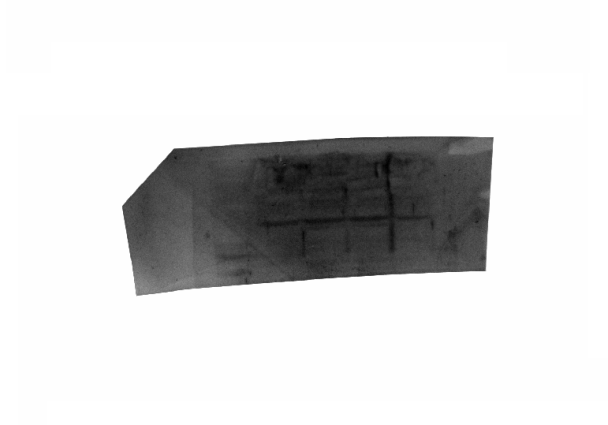


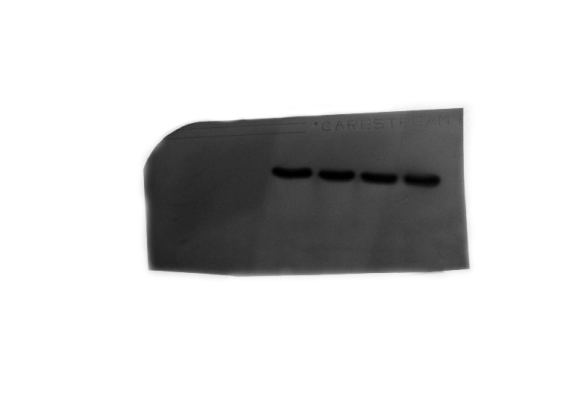


**Figure 10**


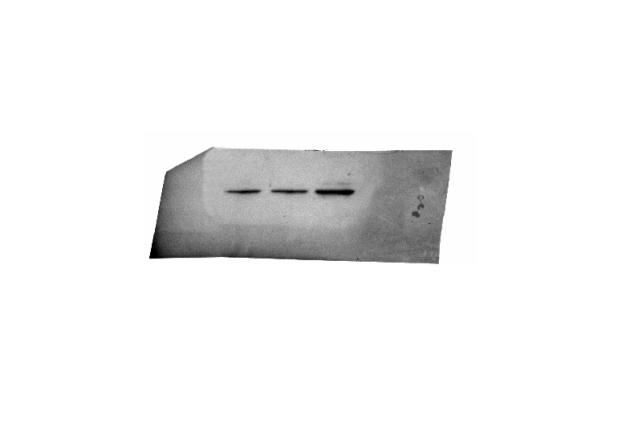

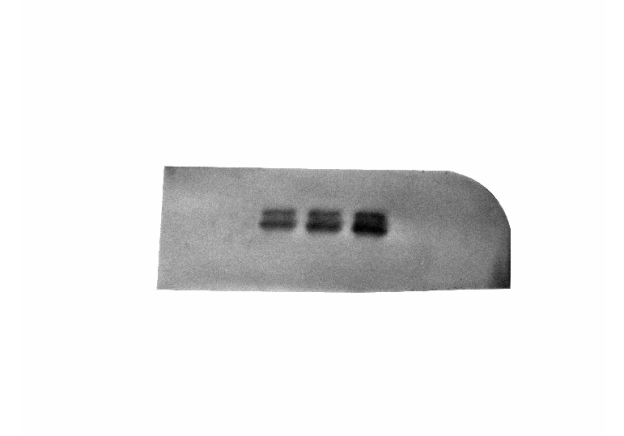


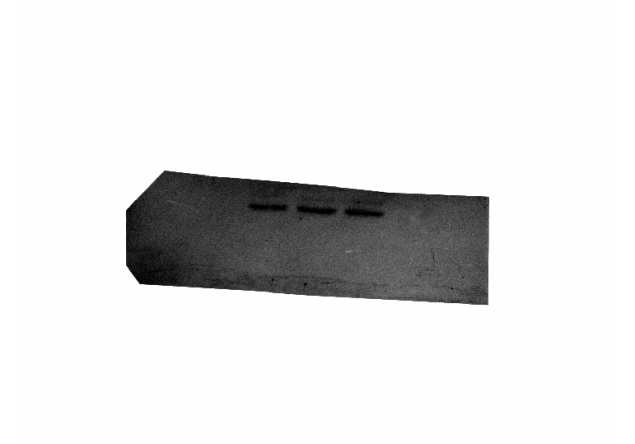

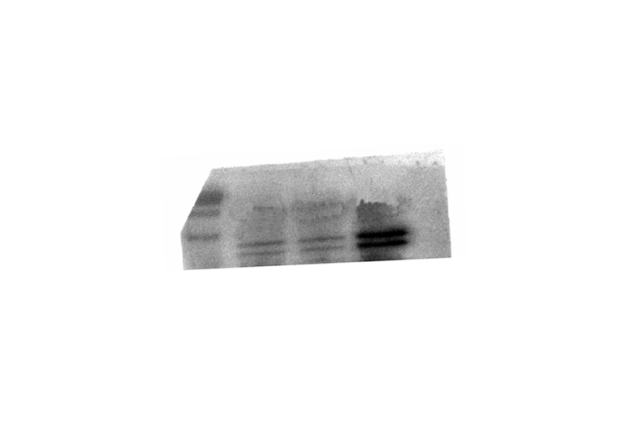


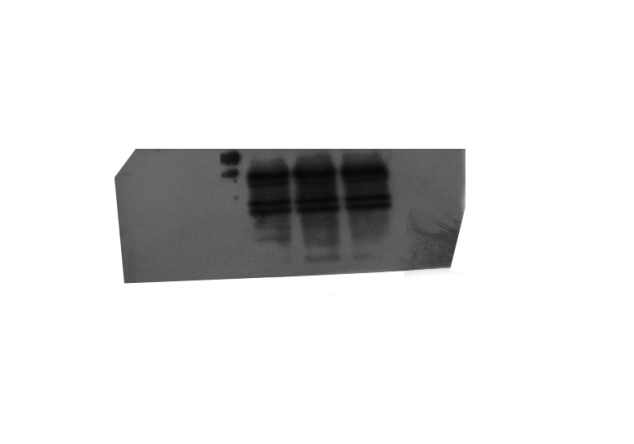

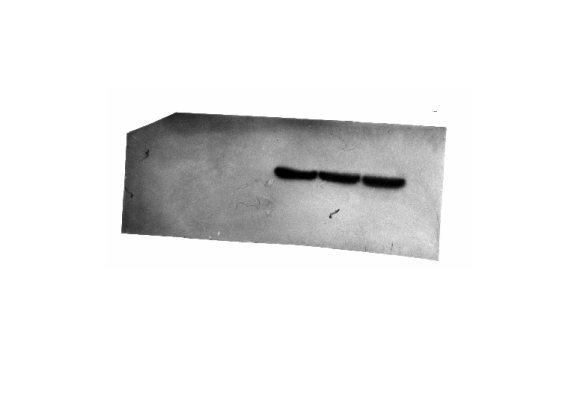

Supplement: Supplementary file 2 — Supplementary file2 (DOCX 1574 KB) [file 12035_2021_2520_MOESM2_ESM.docx]
